# Asymmetric conformations of cleaved HIV-1 envelope glycoprotein trimers in styrene-maleic acid lipid nanoparticles

**DOI:** 10.1038/s42003-023-04916-w

**Published:** 2023-05-18

**Authors:** Kunyu Wang, Shijian Zhang, Eden P. Go, Haitao Ding, Wei Li Wang, Hanh T. Nguyen, John C. Kappes, Heather Desaire, Joseph Sodroski, Youdong Mao

**Affiliations:** 1 State Key Laboratory for Mesoscopic Physics, School of Physics, Peking University, Beijing, China; 2 Peking-Tsinghua Joint Center for Life Science, Peking University, Beijing, China; 3 Department of Cancer Immunology and Virology, Dana-Farber Cancer Institute, Boston, MA USA; 4 Department of Microbiology, Harvard Medical School, Boston, MA USA; 5 Department of Chemistry, University of Kansas, Lawrence, KS USA; 6 Department of Medicine, University of Alabama at Birmingham, Birmingham, AL USA; 7 Birmingham Veterans Affairs Medical Center, Research Service, Birmingham, AL USA; 8 Department of Immunology and Infectious Diseases, Harvard T.H. Chan School of Public Health, Boston, MA USA; 9 Center for Quantitative Biology, Academy of Advanced Interdisciplinary Studies, Peking University, Beijing, China; 10 National Biomedical Imaging Center, Peking University, Beijing, China

**Keywords:** Cryoelectron microscopy, Virus structures

## Abstract

During virus entry, the pretriggered human immunodeficiency virus (HIV-1) envelope glycoprotein (Env) trimer initially transits into a default intermediate state (DIS) that remains structurally uncharacterized. Here, we present cryo-EM structures at near-atomic resolution of two cleaved full-length HIV-1 Env trimers purified from cell membranes in styrene-maleic acid lipid nanoparticles without antibodies or receptors. The cleaved Env trimers exhibited tighter subunit packing than uncleaved trimers. Cleaved and uncleaved Env trimers assumed remarkably consistent yet distinct asymmetric conformations, with one smaller and two larger opening angles. Breaking conformational symmetry is allosterically coupled with dynamic helical transformations of the gp41 N-terminal heptad repeat (HR1_N_) regions in two protomers and with trimer tilting in the membrane. The broken symmetry of the DIS potentially assists Env binding to two CD4 receptors—while resisting antibody binding—and promotes extension of the gp41 HR1 helical coiled-coil, which relocates the fusion peptide closer to the target cell membrane.

## Abstract (web summary)

Cryo-EM structures reveal an asymmetric conformation of the HIV-1 Env trimer suggestive of a default intermediate state important for virus entry and antibody evasion.

## Introduction

The entry of human immunodeficiency virus type-1 (HIV-1), the etiologic agent of AIDS, into host cells is mediated by the envelope glycoprotein (Env) trimer^1,2^. In infected cells, Env is synthesized as a gp160 precursor in the endoplasmic reticulum, where trimerization, disulfide bonding and the addition of high-mannose glycans occur^3,4^. During transport through the Golgi apparatus, a subset of the Env glycans are modified to complex carbohydrates and the Env precursor is cleaved by host furin-like proteases^3,4^. The resulting mature Env, which consists of a gp120 exterior subunit and a gp41 transmembrane subunit in each of the three protomers, is incorporated into budding virions.

During virus entry, gp120 engages the target cell receptors, CD4 and CCR5 (or CXCR4), and gp41 fuses the viral and cell membranes^1,2^. Both spontaneous and CD4-induced transitions of virion Env conformations have been characterized by single-molecule fluorescence resonance energy transfer (smFRET)^5^. Binding to the host receptors drives the metastable (State-1) pretriggered Env trimer into lower-energy conformations along the entry pathway. CD4 binding initially induces the default intermediate state (DIS), an asymmetric trimer in which the CD4-bound protomer is in State-3 and the unbound protomers are in State-2^5–8^. To an extent dependent on HIV-1 strain, Envs can spontaneously sample conformations resembling the DIS in the absence of CD4^5^. Destabilization of the pretriggered (State-1) Env trimer by particular Env amino acid changes or disruption of membrane anchorage can also lead to the DIS-like conformations^6,9–13^. Binding of multiple CD4 molecules generates the State-3 prehairpin intermediate, in which the gp41 heptad repeat 1 (HR1) regions form an extended coiled coil that relocates the hydrophobic gp41 fusion peptides in the target cell membrane^2,14,15^. CCR5/CXCR4 binding triggers further conformational transitions in the gp41 HR1 and HR2 regions leading to the formation of a six-helix bundle that mediates the fusion of the viral and cellular membranes^2,16–18^.

HIV-1 establishes persistent infections in humans and has evolved mechanisms to avoid host immune responses. As the only virus-specific protein on the surface of the virion, Env serves as a target for neutralizing antibodies elicited in infected individuals^19^. Conformational flexibility, heavy glycosylation, high mutability and strain variation of HIV-1 Env contribute to antibody evasion^20^. Most antibodies generated during natural HIV-1 infection are poorly neutralizing, as they fail to bind conserved regions accessible on the cleaved pretriggered (State-1) Env trimer^19–23^. Broadly neutralizing antibodies (bNAbs) generally recognize this pretriggered (State-1) Env conformation but are only sporadically generated during natural infection^19–23^. Soluble stabilized (SOSIP) Env trimers retain many bNAb epitopes, allowing structural characterization of bNAb–Env trimer interactions^20–22,24^. However, to date, SOSIP trimers have not efficiently elicited bNAbs in animals or humans^25–28^. Potentially contributing to this difficulty are differences in antigenicity and glycosylation between SOSIP trimers and membrane Envs^24,29–32^. An smFRET study found that the conformations of individual protomers of the SOSIP trimers resemble State-2 more than the pretriggered (State-1) Env conformation^32^. These results indicate that a detailed structure of the pretriggered (State-1) Env conformation is currently lacking. As State-1 Env represents the major target for bNAbs^5,23^, this gap in knowledge could slow the development of successful vaccines. Moreover, the State-1-to-State-2 transition is modulated by both major classes of gp120-directed small-molecule entry inhibitors, BMS-806 and CD4-mimetic compounds (CD4mcs)^5,12,14^. More detailed information on the relationship between the pretriggered (State-1) Env conformation and the DIS would fill gaps in understanding the process of HIV-1 entry and assist the rational design of entry inhibitors and vaccines.

Here we used cryo-electron microscopy (cryo-EM) to solve the structures of two cleaved full-length HIV-1 Env trimers. We employed several measures designed to preserve the conformations of the native membrane Env during solubilization and purification. The Env trimers were purified directly from cell membranes in styrene-maleic acid lipid nanoparticles (SMALPs), bypassing detergent solubilization^33^. The AD8 and AE2 Envs were derived from the primary HIV-1_AD8_ strain; the AE2 Env variant was modified from the AD8 Env to retain greater stability of the functional pretriggered (State-1) conformation and also contains additional lysine residues to facilitate cross-linking^34^. The Envs were complexed with BMS-806, a small-molecule HIV-1 entry inhibitor that decreases transitions from the pretriggered (State-1) conformation^5,12,14^. We avoided the use of protein ligands that might potentially alter or restrict Env conformation. Despite these measures, the solubilized AD8 and AE2 Envs exhibited differences in antigenicity from the pretriggered (State-1) conformation of the cleaved membrane Env. Nonetheless, the structures of the two cleaved Env trimers in SMALPs are informative.

Compared with a previously studied uncleaved HIV-1_JR-FL_ Env(–) trimer^12^, the cleaved AD8 and AE2 trimers exhibited tighter subunit packing. The cleaved and uncleaved Env trimers exhibited remarkably similar asymmetric conformations despite differences in sequence and preparation variables; this suggests that HIV-1 Env has a natural tendency to assume this default conformation, which is antigenically distinct from the pretriggered (State-1) membrane Env conformation. The opening of the Env protomers in the asymmetric trimers was allosterically coupled with helical transformations of the gp41 HR1_N_ region. We identified the tilting mode of the Env ectodomain with respect to the gp41 membrane-proximal external region (MPER) and transmembrane (TM) region and correlated the direction of tilt with the asymmetric opening of the Env trimer. This study provides candidate structures for the DIS of the membrane HIV-1 Env trimer. Our findings reveal how the asymmetry of this early Env intermediate facilitates gp120 receptor binding, gp41 conformational transitions critical for virus entry, and antibody evasion by HIV-1.

## Results

### Purification and characterization of cleaved full-length AD8 and AE2 HIV-1 Envs

The full-length AD8 Env and its lysine-rich derivative, AE2, which contains changes that favor the functional pretriggered conformation^34^, were expressed in A549 human lung epithelial cells (Supplementary Fig. 1a). Membranes prepared from these cells were used for Env purification. BMS-806, which hinders Env transitions from a pretriggered conformation^5,14^, was added to the membranes and was present throughout the purification of the AD8 and AE2 Envs. Membranes containing the AE2 Env were cross-linked with 3,3’-dithiobis (sulfosuccinimidyl propionate) (DTSSP). The Envs were extracted from the membranes with styrene-maleic acid (SMA) copolymers^33^, purified using the C-terminal six-histidine (His_6_) epitope tag, and counterselected with poorly neutralizing monoclonal antibodies to remove uncleaved Envs^12^. Approximately 76% and 91% of the respective AD8 and AE2 Envs in the purified preparations were cleaved (Supplementary Fig. 1b). Trimers of the DTSSP-cross-linked AE2 Env were evident on non-reducing gels.

### Determination of AD8 and AE2 Env structures by cryo-EM

**Fig. 1:**
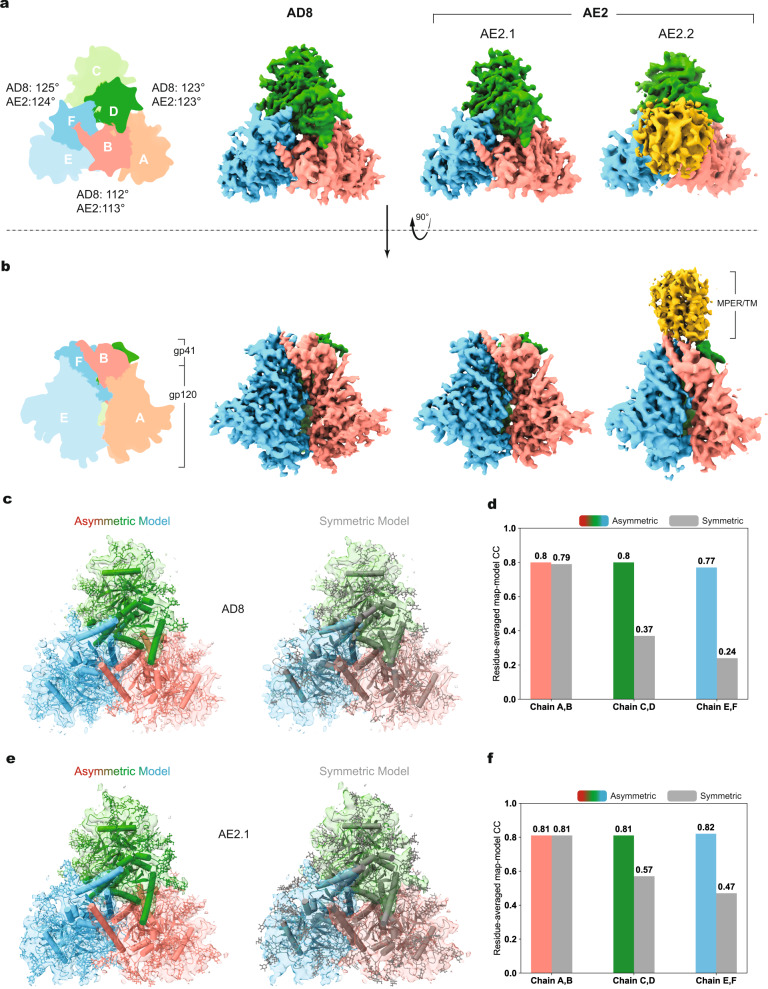
Asymmetric structures of the AD8 and AE2 Env trimers. **a** Views of the AD8, AE2.1, and AE2.2 Env density maps along the trimer axis, from the perspective of the expressing cell/viral membrane. The individual protomers of the Env trimers are colored blue, green, and coral red. The schematic diagram on the left indicates the designations of the gp120 (**a**, **c**, and **e**) and gp41 (**b**, **d**, and **f**) chains and color scheme that will be used throughout the rest of the manuscript. The opening angles of each of the interprotomer interfaces in the AD8 and AE2.1 Env trimers are shown. The density associated with the gp41 membrane-proximal external regions (MPERs) and transmembrane (TM) regions in the AE2.2 map is colored yellow. **b** Side views of the AD8, AE2.1, and AE2.2 Env density maps, with the gp41 subunits at the top and the gp120 subunits at the bottom of the images. **c**, **e** The density maps of the AD8 (**c**) or AE2.1 (**e**) Envs fitted with asymmetric trimer models (left) or pseudo-C3 symmetric models (right) are shown. The structures of the Env protomers in the pseudo-C3 symmetric model are identical to those in the asymmetric model, except that the rotational angles between each pair of protomers was set to 120°. The pseudo-C3 symmetric models were fitted into the density of one protomer (Chains A and B). For all models, only the first mannose residue on each glycan is shown. **d**, **f** Map-model correlation coefficients (CC) for the symmetric and asymmetric models were calculated for each protomer of the AD8 (**d**) or AE2.1 (**f**) Env. The colors of the bars for the asymmetric models correspond to the colors of the chains in the maps and models. The overall residue CC (without glycans) was calculated for each protomer; the CC value shown is the average of the CC values of the three protomers.

We used single-particle cryo-EM to analyze the structures of the AD8 and AE2 Envs. Using a strategy validated in a previous study^12^, we included a substantial fraction of data collected at a highly tilted sample stage, thus alleviating the effect of the orientation preference of the Env trimer particles in cryo-EM reconstruction. Comparison of the 2D class averages for data collected at different tilt angles revealed no obvious difference in image quality when using gold substrate-supported cryo-EM grids for minimizing beam-induced sample movement (Supplementary Fig. 2a–d). Extensive 3D classification of the AE2 data resulted in two 3D maps refined to resolutions of 3.8 Å and 5.0 Å, designated AE2.1 and AE2.2 respectively (Fig. 1a, b, Table 1, and Supplementary Figs. 3 and 4b). The Env ectodomains of the AE2.1 and AE2.2 conformations resembled each other, taking into account the differences in map quality caused by unmatched particle numbers. The AE2.2 map exhibited additional density associated with the gp41 membrane-proximal external region (MPER) and transmembrane (TM) region (Fig. 1a, b). Processing of the AD8 data likewise yielded two Env trimer conformations (Supplementary Fig. 4a); only the major conformation (herein designated AD8) with higher resolution was refined to completion, with an average resolution of 4.1 Å (Fig. 1a, b). All 3D classifications for the final AE2.1, AE2.2, and AD8 maps were conducted without imposing any symmetry constraints; in fact, the imposition of C3 symmetry reduced the estimated resolution or quality of the maps and prevented the reconstructions from achieving resolutions in the near-atomic range.

**Table 1:** Cryo-EM data collection, refinement, and validation statistics.

	HIV-1_AD8_ Env (EMD-28953) (8FAD)	HIV-1_AE2_ Env (EMD-28954) (8FAE)
*Data collection and processing*		
Magnification	105,000	105,000
Voltage (kV)	300	300
Electron exposure (e/Å)	54	52/56/62
Defocus range (μm)	−1.0 to −2.7	−1.0 to −3.0
Pixel size (Å)	0.685	0.825
Symmetry imposed	C1	C1
Initial particle images (no.)	365,824	1,209,493
Final particle images (no.)	37,797	140,035
Map resolution (Å)	4.1	3.8
FSC threshold	0.143	0.143
Map resolution range (Å)	3.6–8	3.6–8
*Refinement*		
Initial model used (PDB code)	7N6U	7N6U
Model resolution (Å)	4.0	3.9
FSC threshold	0.143	0.143
Model resolution range (Å)	3.5–8	3.5–8
Map sharpening B factor (Å^2^)	−80	−80
Model composition		
Non-hydrogen atoms	16,210	17,693
Protein residues	1797	1832
Ligands	145	250
B factors (Å^2^)		
Protein	86.11	184.36
Ligands	133.64	105.59
*R.m.s. deviations*		
Bond lengths (Å)	0.010	0.012
Bond angles (degree)	1.471	1.596
*Validation*		
MolProbity score	2.51	2.55
Clashscore	10.10	8.21
Poor rotamers (%)	3.58	5.71
*Ramachandran plot*		
Favored (%)	89.48	90.57
Allowed (%)	9.57	8.49
Disallowed (%)	0.96	0.94

The quality of the AD8, AE2.1, and AE2.2 density maps allowed atomic modeling and refinement with accuracy to the level of the C_α_ backbone trace and some large side chains (e.g., Trp 112, Arg 579, and Trp 571) (Supplementary Fig. 2e–i). The ectodomains of the AD8, AE2.1, and AE2.2 trimers share an overall topology with each other and with those of existing soluble and membrane HIV-1 Env trimer structures^12,31,35–41^. In all these structures, the gp120 subunits project outwards from the gp41 ectodomains, with the C-terminal portion of each gp41 HR1 region (HR1_C_) participating in the formation of a central three-helix bundle. The ectodomains of SOSIP trimers are truncated after gp41 residue 664, removing the MPER, TM, and cytoplasmic tail^24,31^. These gp41 regions are not well resolved in structures of detergent-solubilized membrane HIV-1 Envs, even after reconstitution into lipid-bilayer nanodiscs^12,39–41^. In the AE2.2 map, low-resolution density associated with the gp41 MPER and TM region was evident (Fig. 1a, b and Supplementary Figs. 3h and 4b). The gp41 cytoplasmic tail was not resolved in either the AD8 or AE2 maps.

Fitting asymmetric Env trimer models into the AD8, AE2.1, and AE2.2 density maps resulted in higher map-model correlations than fitting symmetrical trimer models (Fig. 1c–f). As was seen for the uncleaved, detergent-solubilized Env(–) trimers^12^, the major conformations of the cleaved AD8 and AE2 Env trimers in SMALPs are asymmetric. This asymmetry results from differential rotation and translation of the protomers with respect to the trimer axis. The asymmetric architectures of the AD8, AE2, and Env(–) ectodomains are consistent in terms of the geometric relationship between adjacent gp120 subunits, with two opening angles >120° and one opening angle <120° (Fig. 1a).

### Comparison of the three protomers in the AD8, AE2, and Env(–) trimers

**Fig. 2:**
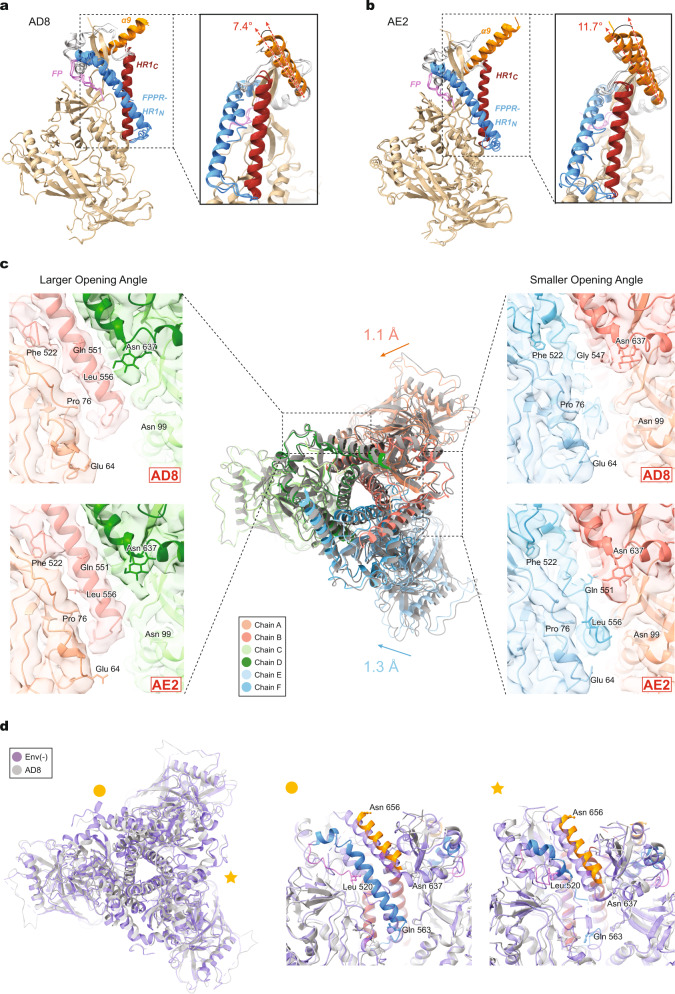
Comparison of Env protomer conformations. **a** Molecular structures of the three protomers of the AD8 Env model were aligned using the gp120 subunits. The gp41 fusion peptide (FP), FPPR + HR1_N_, HR1_C_ and α9 helix are colored violet, blue, red and orange, respectively. The inset shows that the α9 helices in the three protomers rotate through a range of 7.4°. **b** The molecular structures of the three protomers of the AE2.1 Env model, aligned and colored as in (**a**). The largest angle between two α9 helices is 11.7°. **c** The AD8 and AE2.1 Env trimer models were superimposed, based on alignment of one protomer (Chains C and D). In the superposed structures in the center, the AD8 Env model is colored gray and the AE2.1 model is colored according to the chain. The non-aligned protomers of the AE2.1 Env are closer to the trimer axis by 1.1 Å and 1.3 Å, relative to the corresponding AD8 Env protomers. Close-up side views of the density maps and models of an interprotomer interface with a larger opening angle (left) and a smaller opening angle (right) are shown, colored according to the chain. The HR1_N_ region is shown in the close-up views. HR1_N_ is an α-helix in the interprotomer interfaces with larger opening angles, whereas HR1_N_ in the interface with a smaller opening angle is either poorly ordered (for AD8) or a loop (for AE2.1). **d** The AD8 and Env(–) trimer models were superposed, based on alignment of one protomer (Chains C and D). In the left panel, the AD8 Env is in gray and Env(–) in purple. The interprotomer interface with a small opening angle is indicated with a star; one of the interprotomer interfaces with a large opening angle is indicated with a circle. Close-up side views of the interprotomer interfaces with a smaller opening angle (star) and a larger opening angle (circle) are shown in the right panels. In the right panels, the Env(–) model is colored purple; the AD8 Env model is colored gray, except for specific highlighted regions, colored as in (**a**).

The three protomers within the AD8 and AE2 Env trimers exhibit similar folds, with the greatest variation among the protomers occurring in the gp41 subunits. Aligning the gp120 subunits generated interprotomer C_α_ root mean square deviations (RMSDs) of 0.24–0.33 Å for the AD8 trimer (Fig. 2a) and 0.78–0.82 Å for the AE2.1 trimer (Fig. 2b). Asymmetry in these trimers is apparently facilitated by structural rearrangements in the fusion peptide-proximal region (FPPR), HR1_N_ region and α9 helix of the gp41 subunits. The α9 helices in the three aligned protomers of the AD8 Env trimers exhibit rotations of up to 7.4° (Fig. 2a). The extent of α9 helix rotation is even greater, up to 11.7°, among the protomers of the AE2.1 Env trimer (Fig. 2b). The different positioning of the α9 helices reflects asymmetry among the interprotomer interfaces, with the smallest interface placing the α9 helix in one protomer under steric constraints from the adjacent protomer. In this interprotomer interface, there is potential for hydrogen bonding between Thr 538 of the FPPR and either Asn 651 (in AD8) or Gln 652 (in AE2.1) of the α9 helix, supported by strong side-chain density in the maps (Supplementary Fig. 2e, f).

Aligning the AD8 and AE2.1 trimer models using the chain C gp120 subunits demonstrates the similarities in the overall composition and opening angles of these trimers (Fig. 2c). The other two protomers in the AE2.1 Env are translated around 1 Å toward the trimer axis relative to their positions in the AD8 Env, suggesting a more compact AE2.1 trimer structure. For both AD8 and AE2.1 Env trimers, the largest structural differences among the protomers occur in the gp41 FPPR and HR1_N_ regions (residues 534–543 and 544–570, respectively) leading to the central HR1_C_ three-helix bundle. In the two interprotomer interfaces with the larger opening angles, the FPPR and HR1_N_ assume helical conformations (Fig. 2c). By contrast, in the interprotomer interface with the smaller opening angle, HR1_N_ forms a loop that is well resolved in the AE2.1 Env; in the AD8 Env, the density associated with this loop is weaker, likely indicating a higher level of dynamics (Fig. 2c). Interactions such as a potential hydrogen bond between Thr 51 and Glu 560 could stabilize the gp120–gp41 interface in the AE2.1 Env, allowing the conformationally labile HR1_N_ loop to be resolved (Supplementary Fig. 2g).

Alignment of the AD8 Env with the uncleaved HIV-1_JR-FL_ Env(–) trimer again demonstrated similarities in the overall fold of the protomers, opening angles, and asymmetric topology (Fig. 2d). As previously reported^12^, the helicity of the FPPR–HR1_N_ regions in the two Env(–) interprotomer interfaces with the larger opening angles is greater than that in the interprotomer interface with the smaller opening angle. In the former interfaces, the Env(–) FPPR and HR1_N_ helices aligned well with those of the AD8 Env (Fig. 2d). In the latter interface, both Env(–) and AD8 Envs demonstrated less helical and more disordered HR1_N_ elements. Thus, for both uncleaved and cleaved asymmetric Envs, the FPPR–HR1_N_ regions in the two interprotomer interfaces with the larger opening angles exhibited greater helical structure.

### Asymmetry in HIV-1 Env trimer structures

**Fig. 3:**
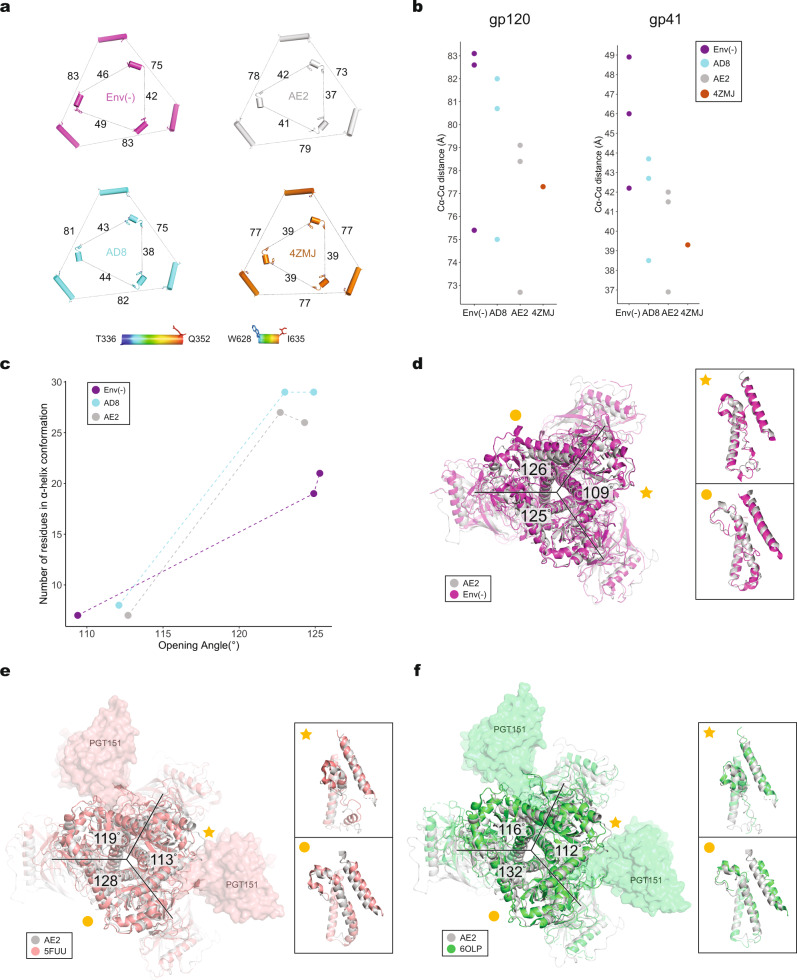
Comparison of trimer geometry among different Env structures. **a** The interprotomer distances (in Å) between selected C_α_ atoms (Thr 336 and Gln 352) of gp120 (outer triangles) and Cα atoms (Trp 628 and Ile 635) of gp41 (inner triangles) are shown for the AD8 and AE2.1 Envs, Env(–) (PDB 7N6U) and an unliganded SOSIP Env trimer (PDB 4ZMJ). **b** The Cα–Cα distances shown in (**a**) are plotted for the gp120 and gp41 subunits. **c** The relationship between helicity of the FPPR–HR1_N_ region and the opening angle of the AD8, AE2.1 and Env(–) trimers is shown. The *x* axis represents the opening angle for each of the interprotomer interfaces, measured in PyMOL. The *y* axis represents the number of residues in an α-helical conformation for the FPPR–HR1_N_ region (residues Ser 534–Val 570) associated with an interprotomer interface. **d** The AE2.1 and Env(–) trimer models were superposed, based on alignment of AE2.1 gp120 (Chain E) with Env(–) gp120 (Chain B). The AE2.1 Env is colored gray and the Env(–) trimer is colored magenta. The opening angles of the Env(–) interprotomer interfaces are shown. **e** Comparison of the AE2.1 Env and the PGT151-bound HIV-1_JR-FL_ EnvΔCT structures (PDB 5FUU). The AE2.1 Env Chain E structure (in gray) is superposed on Chain C of the PGT151-bound HIV-1_JR-FL_ EnvΔCT structure (in salmon), with the PGT151 Fabs shown. The opening angles between the protomers of the PGT151-bound HIV-1_JR-FL_ EnvΔCT trimer are shown. **f** Comparison of the AE2.1 Env and the PGT151-bound HIV-1_AMC011_ EnvΔCT structures (PDB 6OLP). The AE2.1 Env Chain E structure (in gray) is superposed on Chain C of the PGT151-bound HIV-1_AMC011_ EnvΔCT structure (in green), with the PGT151 Fabs shown. The opening angles between the protomers of the PGT151-bound HIV-1_AMC011_ EnvΔCT trimer are shown. **d**–**f** close-up side views of the interprotomer interfaces with a smaller opening angle (star) and a larger opening angle (circle) are shown in the insets. The close-up views show the gp41 fusion peptide, FPPR, HR1_N_ and HR1_C_ regions from the superposed protomers and the α9 helix from the adjacent protomer.

We compared the topology of the Env(–), AD8, and AE2 Env trimers with each other and with those of other HIV-1 Env trimer structures. The interprotomer distances between arbitrarily chosen atoms on the outer surface of gp120 and gp41 provide a measure of trimer geometry (Fig. 3a, b). Using these atoms, the symmetric HIV-1_BG505_ SOSIP trimer exhibits gp120 and gp41 interprotomer C_α_–C_α_ distances of 77 and 39 Å, respectively. The interprotomer C_α_–C_α_ distances increase progressively in the AE2, AD8, and Env(–) trimers, indicating the following rank order of trimer compactness: AE2 > AD8 > HIV-1_JR-FL_ Env(–). These differences in trimer compactness are potentially influenced by the degree of Env cleavage as well as preparation-dependent variables (including HIV-1 strain of origin, stabilizing Env changes, cross-linking, etc.) (Supplementary Fig. 1a).

Regardless of differences in compactness, the Env(–), AD8, and AE2 Env trimers demonstrated remarkably similar asymmetric topologies. Each of these Env trimers has two approximately equal long sides and a short side (Fig. 3a). All three Envs have two opening angles of 123–126° and one opening angle of 109–113°. The conformation of the FPPR–HR1_N_ region is highly correlated with the opening angle of the interprotomer interface in which the FPPR and HR1_N_ reside (Fig. 3c). For interfaces with opening angles greater than 120°, most of the FPPR and HR1_N_ residues reside in an α-helix. By contrast, for interfaces with opening angles less than 120°, a substantial number of HR1_N_ residues sample a loop conformation. These relationships apply to both cleaved and uncleaved Envs, although the number of FPPR–HR1_N_ residues in α-helical form associated with the two larger opening angles is somewhat lower for the uncleaved Env(–) trimer^12^ (Fig. 3c, d). This may reflect the greater flexibility of the uncleaved Env(–) and could influence the reversibility of Env trimer opening^12^. The consistency with which cleaved and uncleaved Env(–) trimers display these asymmetric conformations in the absence of protein ligands (antibodies or receptors) suggests that the observed structures represent stable conformations preferentially assumed by HIV-1 Envs extracted from their membrane environment.

We compared the structures of the AD8, AE2, and Env(–) trimers with structures of other asymmetric HIV-1 Env trimers complexed with bNAb Fabs. In the latter examples, trimer asymmetry is likely induced by Fab binding. We aligned a single protomer of the AE2.1 trimer with those of detergent-solubilized HIV-1_JR-FL_ EnvΔCT (PDB: 5FUU) and HIV-1_AMC011_ EnvΔCT (PDB: 6OLP) trimers complexed with PGT151 Fabs (Fig. 3e, f). The PGT151 bNAb recognizes a hybrid epitope composed of a gp120 glycan at Asn 88 and the gp41 N-terminus, and binds with a stoichiometry of two Fabs per Env trimer^39^. We also aligned an AE2.1 Env protomer with that of the detergent-solubilized HIV-1_AMC011_ Env trimer purified from cell membranes in a complex with the PGT145 Fab (PDB: 6NIJ)^31^ (Supplementary Fig. 5a). The PGT145 bNAb recognizes a glycan-dependent V2 quaternary epitope at the Env trimer apex, and binds with a stoichiometry of one Fab per Env trimer^31^. The asymmetry of the bNAb–Env trimer complexes differ from that of the AE2.1 Env. The bNAb-bound Env trimers have one long side and two shorter sides, with one opening angle >120° and two opening angles <120° (Fig. 3e, f and Supplementary Fig. 5b). Thus, the asymmetry of the PGT151- and PGT145-bound cleaved Env trimers differs from that of the cleaved AD8 and AE2 Env trimers and from that of the uncleaved Env(–) trimer^12^.

Despite the significant variation in the asymmetric topology of the antibody Fab-bound and -free HIV-1 Env trimers, the relationship between the opening angle and the helicity of the HR1_N_ region was generally maintained (Supplementary Fig. 5b). In the PGT151-bound protomers associated with smaller opening angles, HR1_N_ exhibits more flexible loop elements; by contrast, at the largest interprotomer interface where no antibody Fab is bound, the HR1_N_ helix is well formed and oriented similarly to the HR1_N_ helices located in the larger interprotomer interfaces of the AE2.1 and AD8 Envs (Fig. 3e, f). Likewise, a high degree of HR1_N_ helicity is found in the gp41 subunit (chain B) associated with the largest opening angle of the PGT145-bound Env timer (Supplementary Fig. 5a). These observations suggest a close allosteric coupling between trimer opening and the formation of a stable HR1_N_ helix in the gp41 subunit located in the open interprotomer interface.

### Potential influence of Env trimer asymmetry on CD4 binding

**Fig. 4:**
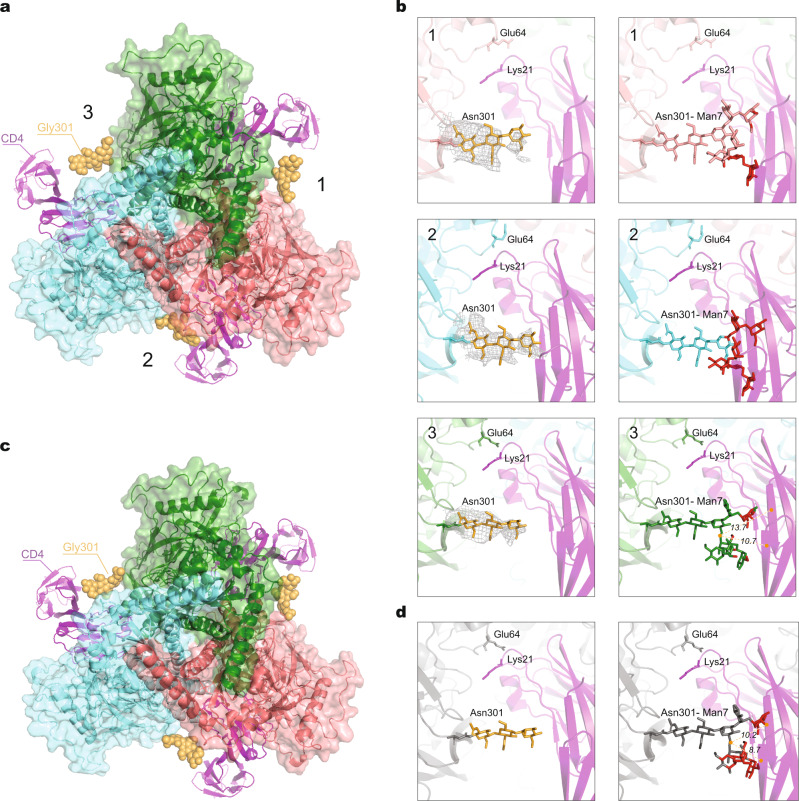
Comparison of CD4 binding to asymmetric and pseudo-symmetric Env trimers. **a** The gp120 chain of a gp120–CD4 complex (PDB 3JWO) was superposed on all three gp120 subunits of the asymmetric AE2.1 Env trimer (colored according to chains). The docked two-domain CD4 molecules are shown as magenta ribbons. Interprotomer interfaces 1 and 3 have larger opening angles; interprotomer interface 2 has a small opening angle. In interface 2, CD4 binding to the AE2.1 Env brings it into very close proximity to the glycans on Asn 301 modeled on the basis of the observed density map. **b** The panels in the left column show Asn 301 glycan structures from the AE2.1 model, with real cryo-EM density surrounding the yellow glycan residues. The panels in the right column show Asn 301 Man7 glycans with additional terminal mannose residues constructed in silico, colored the same as their associated Env protomers. At each interprotomer interface, any atoms on the Asn 301 glycans that are within 4 Å of CD4 are colored red. At interprotomer interface 3, the distance between the center of mass of the whole Man7 glycan and that of one β-sheet (Lys 2–Gly 6) on CD4 is 13.7 Å; the distance for the other β-sheet (Lys 166–Glu 169) on CD4 is 10.7 Å**. c** The gp120 chain of a gp120–CD4 complex (PDB 3JWO) was superposed on all three gp120 subunits of a C3 pseudo-symmetric AE2.1 Env trimer. This pseudo-symmetric AE2.1 Env structure is identical to the one used in Fig. 1e. CD4 is closer to the Asn 301 glycan on the adjacent protomer for the pseudo-symmetric AE2.1 trimer compared with its binding to the more open interfaces of the asymmetric AE2.1 trimer; however, no clash is encountered with the pseudo-symmetric trimer. **d** The left panel shows the Asn 301 glycan structure on one interprotomer interface of the C3 pseudo-symmetric AE2.1 Env trimer; the right panel shows the Asn 301 Man7 glycan with additional terminal mannose residues constructed in silico. Any atoms on the Asn 301 glycan that are within 4 Å of CD4 are colored red. The distance between the center of mass of the whole Man7 glycan and that of one β-sheet (Lys 2–Gly 6) on CD4 is 10.2 Å; the distance for the other β-sheet (Lys 166–Glu 169) on CD4 is 8.7 Å.

Studies of HIV-1 Env trimer stoichiometry indicate that the binding of at least two CD4 molecules promotes efficient virus entry^42^. To evaluate the potential impact of the cleaved Env trimer’s quaternary structure on CD4 binding, we rigidly fit a gp120 core structure bound to two-domain CD4^43^ into the three gp120 subunits of the AE2.1 trimer structure, including the glycans that were rigorously built according to the density map (Fig. 4a). In the interprotomer interface with the smallest opening angle (interface 2 in Fig. 4a, b), CD4 encounters a tight fit with the Asn 301 glycan on the adjacent protomer. Steric limitations for CD4 binding were not evident on the other, larger interprotomer interfaces (Fig. 4b, left panels). Because the atomic models of partial glycan were conservatively constructed from noise-free density maps, the actual, complete glycan tree on Asn 301 contains additional sugar residues emanating from the trimannosyl core. Therefore, we added terminal sugar residues to the experimentally determined atomic model to simulate Man7 carbohydrate chains at Asn 301 in silico and repeated our analysis. With these more realistic glycan chains, unavoidable clashes with a β-sheet (Lys 2–Gly 6) on CD4 domain 1 and a β-sheet (Lys 166–Glu 169) on CD4 domain 2 occurred in the interprotomer interface with the smallest opening angle; the other two interprotomer interfaces were able to accommodate CD4 with fewer, potentially avoidable steric clashes (Fig. 4b, right panels).

To assess the potential role of trimer asymmetry in binding CD4, we imposed C3 symmetry in the final round of refinement of the AE2.1 dataset, thereby generating a pseudo-symmetric Env trimer structure. We fit the structure of the gp120 core bound to two-domain CD4^43^ into this pseudo-symmetric Env trimer model in the same manner as described above (Fig. 4c). In this case, the glycans that were experimentally built based on the density maps were located at least 4 Å away from domains 1 and 2 of CD4 (Fig. 4d, left panel). However, for the more complete Man7 glycans modeled in silico, a high potential for clashes of the terminal sugar residues with CD4 was predicted (Fig. 4d, right panel). Thus, the sampling of asymmetric Env trimer structures like those seen in the AD8 and AE2 Envs could reduce steric hindrance to CD4 binding imposed by the Asn 301 glycan on the adjacent Env protomer. Relieving these steric constraints in the two interprotomer interfaces with larger opening angles would facilitate the binding of two CD4 molecules, a requisite for triggering virus entry^42^.

### Relationship between Env trimer tilting and asymmetric opening

**Fig. 5:**
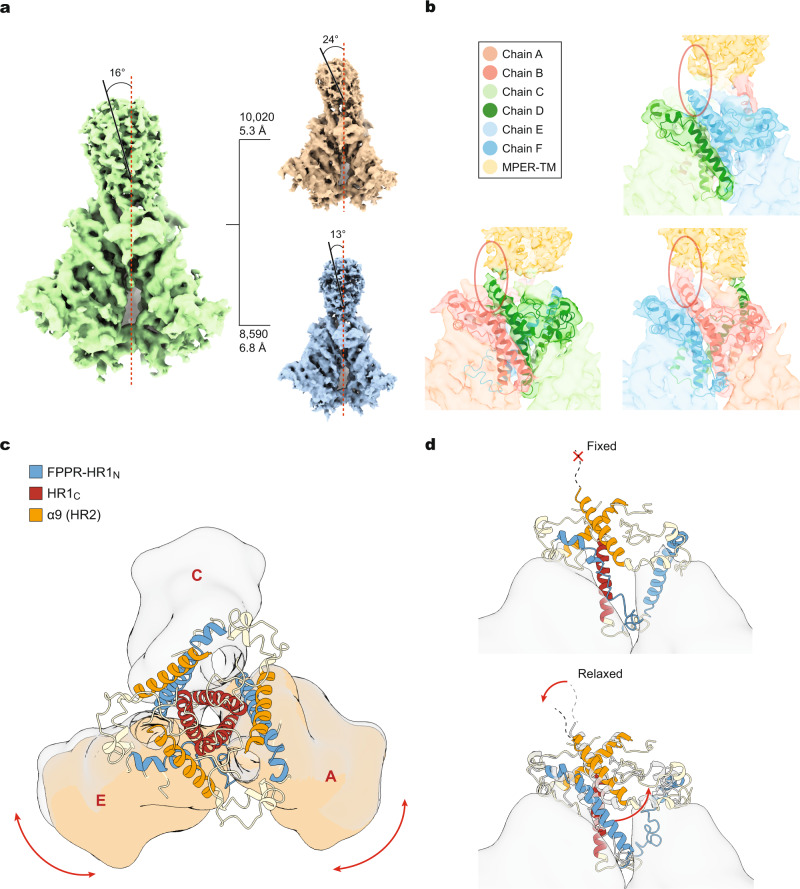
Env trimer tilting and Env ectodomain conformation. **a** The cryo-EM density map of the AE2.2 Env (green) shows the 16° tilt of the ectodomain with respect to the gp41 MPER and TM. Hierarchical 3D classification indicates conformational flexibility of this tilted state. The red dashed line represents the trimer axis; the black solid line represents the axis of a cylinder modeled into the MPER density. Two additional subclasses are shown, with the particle number used to generate each map and the estimated resolution. **b** The three interprotomer interfaces of the AE2.2 Env trimer are shown, with the chains colored according to the key. **c** A schematic diagram showing the spatial relationships of the FPPR–HR1_N_ (blue), HR1_C_ (red) and α9 helices (orange) on the AE2.1 Env trimer. The gp120 subunits are shown as a low-pass filtered surface. The two gp120 subunits flanking the interprotomer interface with the smallest opening angle are shaded brown. **d** A hypothetical model explaining how relaxation of the α9 helix regulates the formation of the DIS. The gp41 components are colored as in (**c**). When α9 is fixed by interaction with the MPER/membrane, its bonding to FPPR preserves the loop conformation of the associated HR1_N_ region (upper panel). When the interaction of α9 with the MPER is relaxed (e.g., by solubilization of Env, by Env tilting), α9 is free to interact with the nascent HR1_N_ helix (lower panel). The α9–HR1_N_ helix interactions stabilize the opening of the protomers to create the interfaces with opening angles of 123°–125°.

Although the AE2.2 map exhibits stable density corresponding to the gp41 MPER and TM regions, secondary structural features in these elements are buried in noise. Performing local refinement of the masked MPER-TM density did not result in better resolution. Nonetheless, the conical MPER-TM density in the AE2.2 Env map has a major axis that deviates approximately 16° from the trimer axis of the AE2.2 Env ectodomain (Fig. 5a). We conducted extensive 3D classification of the AE2.2 data using two generally used software programs, RELION 3.0 and CryoSPARC, for cross-validation. Two subclasses with slightly different tilt angles were obtained: one map, built from 10,020 particles, exhibited a tilt angle of approximately 24°; the other map built from fewer particles exhibited a tilt angle of approximately 13° (Fig. 5a). Although the resolution of these subclasses is limited by the lower particle number compared with the AE2.2 map, all three maps share consistent features that allow several conclusions. First, assuming that the major axis of the MPER-TM density is orthogonal to the membrane, the Env ectodomain tilts towards the interprotomer interface with the smaller opening angle. Second, although the α9 helix and MPER are adjacent in the gp41 primary sequence, in the maps, the C-terminal ends of the three α9 helices exhibit different extents of connectivity with the MPER density (Fig. 5b). The α9 helix at the interprotomer interface with the smaller opening angle exhibits the most extensive connection with the MPER. The tilt of the Env trimer is expected to shorten the distance between this closed interface and the membrane, allowing better maintenance of the α9-MPER interaction for the associated protomer. The reciprocal increase in distance from the membrane for the protomers associated with the larger opening angles is apparently more disruptive of α9-MPER interactions, with weaker density at the α9-MPER junctions. Third, Env tilting appears to be allosterically coupled to a coordinated series of transformations that stabilize an asymmetric conformation of the Env trimer ectodomain (Fig. 5c, d). On the two interfaces with larger opening angles, the movement away from the MPER/membrane frees the associated α9 helices to interact with the nascent HR1_N_ helices on the adjacent protomers (Fig. 5d, lower panel). The transition of the HR1_N_ region, whose structure in the pretriggered (State-1) conformation is not yet known, into an α-helix provides further stability to the two open interprotomer interfaces. On the interprotomer interface with the smaller opening angle, the α9 helix is fixed in position by its association with the adjacent MPER. This promotes the interactions of the α9 helix with FPPR residues that preserve the loop conformation of the adjacent HR1_N_ region; the observed potential hydrogen bond between Thr 538 in the FPPR and Asn 651 in α9 (Supplementary Fig. 2f) is indicative of interactions between these elements that maintain the conformation of the closed trimer interface. Thus, the coordinated interactions of α9, FPPR and HR1_N_ serve to stabilize the interprotomer interfaces of the asymmetric trimer.

### Env glycosylation

The density associated with N-linked glycosylation was well preserved in the AD8 and AE2 maps, allowing modeling of all peptide-proximal glycans with the exception of those on Asn 136, 141, 142, 411, and 463 (Supplementary Fig. 6). We did not detect significant asymmetry in the glycan structures added to the AE2.1 Env trimer; in particular, the marked differences in the carbohydrate density at Asn 88 and Asn 356 detected by a cryo-electron tomography study of HIV-1 virus-like particles^44^ were not seen. Our results are consistent with the glycan modification of a symmetric Env precursor.

The glycosylation profile of the AE2 Env in the absence of BMS-806 was determined by mass spectrometry (Supplementary Fig. 7). Despite some strain-dependent differences in potential N-linked glycosylation sites, the glycosylation profile of the AE2 Env was generally similar to that of the previously characterized HIV-1_JR-FL_ Env(–) glycoprotein^12^. Exceptions were the carbohydrates added to Asn 156, Asn 339, and Asn 362, which were modified by 50–60% processed glycans in Env(–)^12^ but exclusively by high-mannose glycans in the AE2 Env. A study of wild-type HIV-1 Env on virions^30^ also detected only high-mannose carbohydrates at these asparagine residues. These results may reflect real differences between cleaved Envs like AE2 and uncleaved Envs, which are more conformationally dynamic^9,12^ and potentially more accessible to carbohydrate-modifying enzymes.

### BMS-806 binding

In the AD8 and AE2.1 maps, densities of approximately equal intensities corresponding to BMS-806 are evident in all three protomers (Supplementary Fig. 8). The position and orientation of BMS-806 are consistent with those in crystal structures of symmetric SOSIP complexes^45^. These findings argue against the hypothetical possibility that incomplete occupancy of the three Env protomers by BMS-806 results in the observed trimer asymmetry.

### Antibody recognition of asymmetric Env trimers

**Fig. 6:**
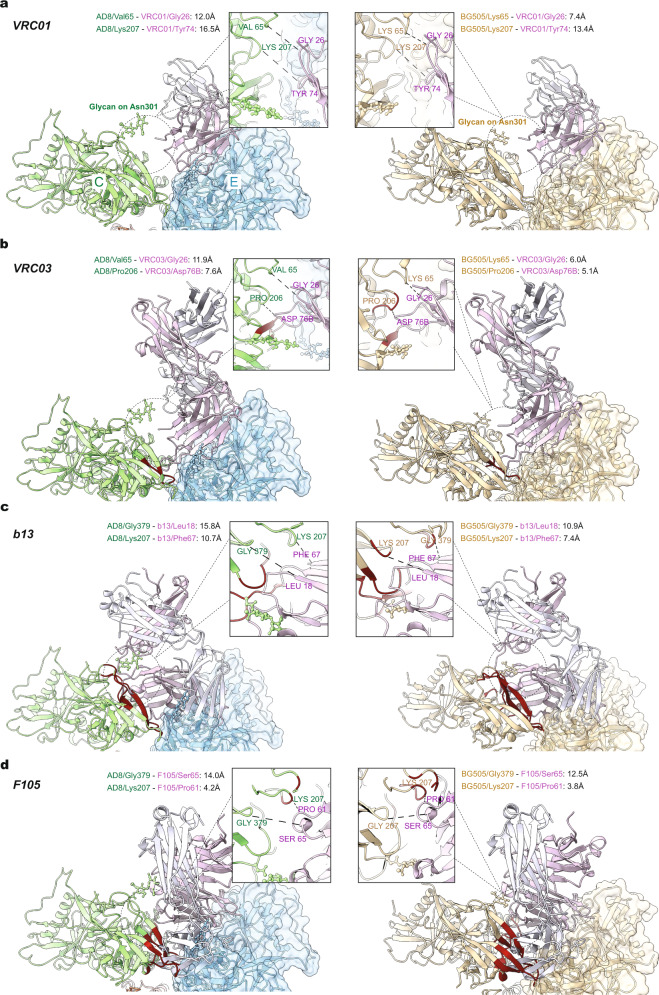
Antibody docking on a symmetric Env trimer and the more open interface of an asymmetric Env trimer. Models of antibody-bound complete Env trimers or subunits were aligned with the asymmetric AD8 trimer model or the symmetric HIV-1_BG505_ SOSIP trimer model (PDB 4ZMJ), according to the gp120 chain. The gp120 of the Env–antibody complex was aligned to Chain E gp120 of the AD8 Env trimer that, along with Chain C gp120, flank an interprotomer interface with a larger opening angle (124°). AD8 models (Chain E is blue and Chain C is green) are on the left; HIV-1_BG505_ SOSIP (wheat) models are on the right. The antibody heavy and light chains are colored with different shades of purple to allow them to be distinguished. The Env protomer bound by the antibody is shown with a molecular surface, and conflict residues in the adjacent Env protomer are shown in red. Conflict residues are defined as containing backbone atoms within 4 Å of any antibody atom. Details of the region of closest approach of antibody and the adjacent Env protomer are shown in the insets. The distances shown are measured between C_α_ atoms of selected residues in ChimeraX^61^. The CD4BS antibodies include: **a** VRC01 (PDB 5FYK); **b** VRC03 (PDB 3SE8); **c** b13 (PDB 3IDY); **d** F105 (PDB 3HI1). VRC01 and VRC03 are CD4BS bNAbs, whereas b13 and F105 are CD4BS pNAbs.

To evaluate the potential impact of the asymmetry observed in the AD8 and AE2 Env trimers on antibody binding, we docked several Env–antibody complex structures onto models of the asymmetric AD8 trimer and the symmetric HIV-1_BG505_ SOSIP trimer. For the AD8 Env, docking was performed on a protomer (Chain E) flanking a more open interface. In general, bNAbs were able to be docked onto both the AD8 and SOSIP trimers with no or minimal clashes, whereas poorly neutralizing antibodies (pNAbs) encountered severe clashes with the adjacent protomer (Fig. 6 and Supplementary Fig. 9). Consistent with the influence of the angle of approach on the neutralizing potency of antibodies against the CD4-binding site (CD4BS)^21–23,46^, the degree of the clash between the CD4BS antibody and the adjacent protomer was inversely correlated with antibody neutralizing potency (Supplementary Table 1). Although the distances between the antibody and gp120 residues on the adjacent protomer were consistently greater for the asymmetric AD8 trimer than for the symmetric SOSIP trimer, the overall ranking of antibodies with respect to the degree of the clash was similar for the two trimers.

The above docking study identified one CD4BS antibody, b12, that is predicted to encounter protein clashes with the adjacent Env protomer on the symmetric SOSIP trimer but not on the asymmetric AD8 trimer (Supplementary Table 1). The b12 bNAb is less potent and broad than the other CD4BS bNAbs studied here and exhibits only modest neutralizing potency against viruses with the AD8 and AE2 Envs (Supplementary Table 2). Even at 20 µg/ml, b12 did not neutralize 90% of AE2 virus infection; by contrast, VRC01 and VRC03 achieved 90% neutralization of the AE2 virus at less than 3 µg/ml. The AE2 virus was completely resistant to neutralization by the pNAbs 19b, 39F and b6, as expected^34^. Thus, b12 neutralizes the AD8 and AE2 viruses at an intermediate level of potency and is predicted to be sensitive to Env trimer asymmetry.

### Ligand binding of cell-surface and solubilized Envs

To test the predictions of the above docking study, we evaluated the ability of sCD4-Ig and a panel of bNAbs and pNAbs to recognize the untreated and BMS-806 treated, DTSSP-cross-linked AE2 Env on the cell surface and after solubilization in SMALPs (Supplementary Fig. 10). With only a few exceptions, bNAbs recognized the untreated cleaved AE2 Env on the cell surface, whereas pNAbs did not. Among the bNAbs tested, b12 was exceptional in failing to recognize the cleaved Env efficiently. Among the pNAbs, b6 and 39F weakly recognized the cleaved Env, which indicates some flexibility in the AE2 Env related to the gp120 CD4BS and V3. The sCD4-Ig precipitated the gp120 glycoprotein but not gp41, suggesting that after sCD4-Ig bound the AE2 Env, some induction of gp120 shedding may have occurred under these conditions. Treatment with BMS-806 and DTSSP reduced recognition of cleaved cell-surface AE2 Env by b6, 39F and sCD4-Ig. Most bNAbs recognized the cleaved AE2 Env after treatment with BMS-806 and DTSSP. Recognition of the BMS-806/DTSSP-treated, cleaved AE2 Env by b12 was undetectable, as was seen for the untreated cleaved Env. Weaker recognition of the BMS-806/DTSSP-treated, cleaved Env by VRC03, PG9 and PGT145 likely resulted from modification of key lysine residues in the epitopes^31,46^. We conclude that the untreated cleaved AE2 Env on the cell surface exhibits an antigenic profile consistent with a pretriggered (State-1) conformation. The b12 antibody, predicted to discriminate between symmetric and asymmetric Env trimers, recognized the cleaved AE2 cell-surface Env inefficiently. Some low-level flexibility of the cell-surface Env, reflected in recognition by the b6 and 39F pNAbs, was apparently eliminated by BMS-806 and DTSSP treatment.

The BMS-806/DTSSP-treated AE2 Env–SMALPs were prepared without counterselection with pNAbs to allow evaluation of antibody binding to cleaved and uncleaved Envs in the preparation. All of the bNAbs, including b12, recognized the cleaved Env–SMALP complexes, as did sCD4-Ig. Recognition of the cleaved Env–SMALP complexes by the 10E8.v4 antibody was more efficient than recognition of the cell-surface Env. By contrast, of the pNAbs, only the b6 CD4BS antibody, the 39F anti-V3 antibody and the anti-gp41 F240 antibody exhibited weak recognition of the cleaved Env–SMALPs. The BMS-806-treated, DTSSP-cross-linked AE2 Env in SMALPs generally retains an antigenic composition resembling but not identical to that of the untreated cell-surface AE2 Env. Notably, the b12 antibody recognized the cleaved AE2 Env–SMALP complexes efficiently, consistent with the prediction of the docking study that the asymmetry of these trimers would allow b12 binding. The exposure of the CD4-binding site and some CD4BS and V3 pNAb epitopes indicates that SMA solubilization introduces some flexibility into the BMS-806/DTSSP-treated membrane AE2 Env.

## Discussion

In this work, we provide structures of full-length, cleaved HIV-1 Envs in SMALPs. Importantly, the cleaved AD8 and AE2 Env structures were solved in the absence of protein ligands, revealing a natural tendency of the solubilized Env trimers to assume consistent asymmetric conformations. Based on the observed differences in antigenicity, we suggest that these asymmetric Env trimers are distinct from the presumably symmetric, pretriggered (State-1) Env anchored in cell or virion membranes. The symmetry of the pretriggered Env trimer is supported by previous cross-linking studies^4^ and by the poor recognition of the cleaved AE2 membrane Env by the b12 bNAb, the binding of which is predicted to be restricted by Env trimer symmetry. Apparently, detergent-free extraction, addition of BMS-806, and cross-linking of the lysine-rich, State-1-stabilized AE2 Env were not sufficient to nullify completely the conformationally disruptive effects of the removal of Env from its membrane environment^12,13^. The recognition of the cleaved Envs in SMALPs by the b12 bNAb is consistent with a loss of Env trimer symmetry upon solubilization. Despite the conformational changes in Env resulting from its extraction from the membrane, the measures we employed preserved membrane-proximal Env interactions in a sufficient number of Env–SMALP complexes to populate the AE2.2 class. Analysis of the AE2.2 Env structures demonstrates that Env tilting in the membrane is not random but rather is coupled to defined asymmetric changes in the Env ectodomain. These changes include opening two of the angles between Env protomers and restructuring of the gp41 FPPR, HR1_N_ and α9 regions. The formation of long HR1_N_ α-helices and their interactions with α9 helices released from association with the MPER stabilize the open interprotomer interfaces. These stabilizing interactions create a local energy well for the asymmetric Env trimer near that of the pretriggered (State-1) Env conformation, with spontaneous and CD4-induced transitions between these conformational states determined by the activation energy barrier between them.

**Fig. 7:**
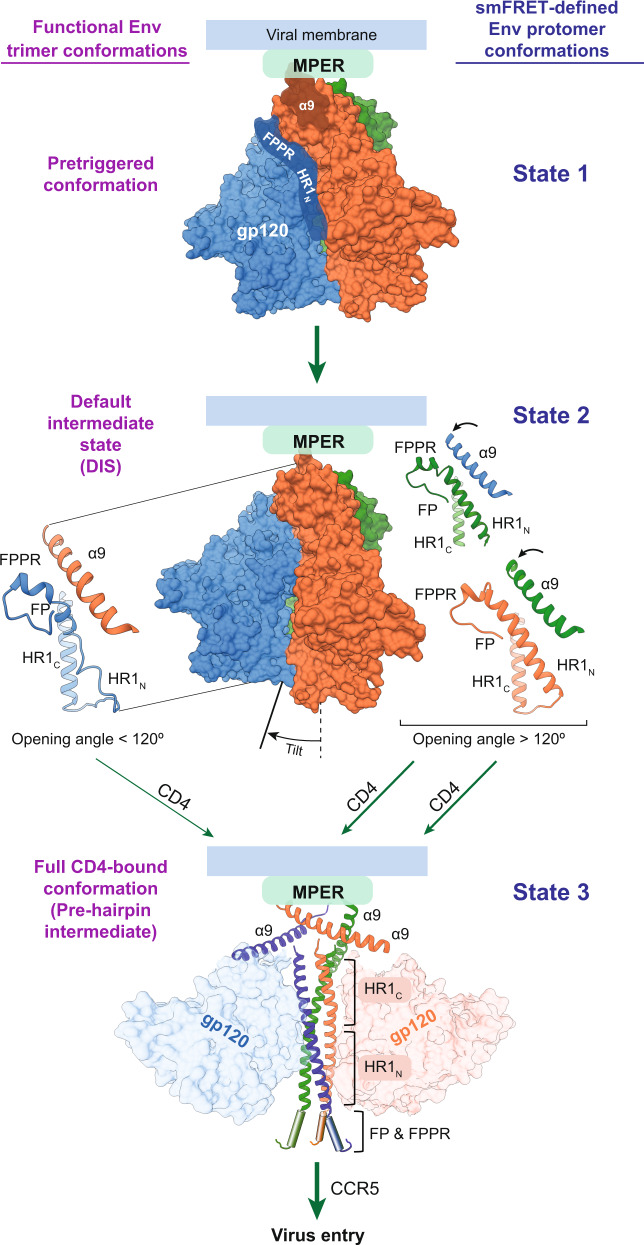
A model involving an asymmetric HIV-1 Env trimer in virus entry. A model of the early steps in HIV-1 entry is shown, with the Env protomers colored as in Fig. 1 (MPER, gp41 membrane-proximal external region; α9, gp41 region spanning residues 628-664; FP, gp41 fusion peptide; FPPR, gp41 fusion peptide-proximal region; HR1_N_ and HR1_C_, gp41 heptad repeat N- and C-terminal regions, respectively). The names of the functional Env trimer conformations are shown to the left of the figures, and the smFRET-defined conformations of the Env protomers are shown to the right of the figures^5–7^. In the pretriggered Env conformation, the close association of the gp41 α9 region with the MPER hypothetically modulates the interaction between α9 and FPPR on the neighboring protomer. In turn, the interaction of FPPR and the adjacent HR1_N_ region with the gp120 inner domain contributes to the maintenance of the pretriggered conformation. Spontaneous transitions between the pretriggered conformation and the asymmetric default intermediate state (DIS) are governed by HIV-1 strain-dependent variables^6,10,11,34^. Tilting of the DIS Env in the membrane is allosterically coupled to asymmetric displacement of the α9 helices, an increase in two of the opening angles between the protomers, and transitions of the associated HR1_N_ regions into helical conformations. In the asymmetric DIS, two protomers can bind CD4 with less steric hindrance and have more helical HR1_N_ regions. Thus, the DIS is predisposed to rearrange into the full CD4-bound conformation (the prehairpin intermediate), where the newly formed HR1_N_ helices extend the HR1_C_ coiled coil, relocating the fusion peptide (FP) closer to the target cell membrane^15^. Binding the second receptor, CCR5, permits the prehairpin intermediate to form the six-helix gp41 bundle that mediates membrane fusion and virus entry. The six-helix bundle is composed of the HR1 coiled coil (HR1_N_ + HR1_C_) and the HR2 helices, which include the α9 helix^16–18^.

The AD8 and AE2 Env trimer structures reported here are good candidates for the default intermediate state (DIS), an obligate Env conformation between the pretriggered (State-1) Env conformation and the full CD4-bound (State-3) conformation on the virus entry pathway^5–7,9,32^ (Fig. 7). The exact nature of the DIS is unknown, but has been shown by smFRET analysis to comprise some protomers in a State-2 conformation^5–7^. The conformations of the individual AD8 and AE2 Env protomers resemble that of soluble SOSIP Env trimers, which have been suggested to assume a State-2-like conformation^32^. Like the AD8 and AE2 Env trimers in SMALPs, the DIS can be asymmetric, at least when initially induced from State-1 by the engagement of a single CD4 molecule^7^. CD4 binding to one Env protomer is thought to release the State-1-stabilizing restraints on the two unbound protomers, which then assume a default (State-2) conformation. The default nature of the State-2 Env conformation is supported by observations that destabilization of State-1 by particular Env amino acid changes or detergent solubilization also lead to increased sampling of State-2^6,9–13^. As expected for a default state that appears upon destabilization of the pretriggered Env, the solubilized AD8 and AE2 Env trimers as well as the Env(−) trimer^12^ exhibit consistent asymmetric conformations, despite differences in sequence and preparation variables (Supplementary Fig. 1a).

The Env trimers on primary HIV-1 virions, although predominantly in a pretriggered (State-1) conformation, spontaneously sample State-2 conformations^5^. Spontaneous sampling of asymmetric trimer conformations like those of the AD8 and AE2 Env observed in our study could facilitate HIV-1 entry into target cells (Fig. 7). The stabilizing effects of the FPPR, HR1_N_ and α9 rearrangements, which are allosterically coupled to Env trimer opening, predispose the presumably symmetrical pretriggered (State-1) Env trimer to assume an asymmetric trimer topology with two opening angles of 123–125° and one opening angle of 112–113°. This asymmetry can facilitate Env trimer binding to two CD4 molecules, the number of receptor-binding events required to propagate subsequent entry-related Env conformational changes^42^. Formation of the HR1_N_ helix simultaneously disassembles the currently unknown conformation of this gp41 element in the pretriggered state, where it is thought to contribute to the non-covalent association of gp120 with the Env trimer^47,48^. The HR1_N_ helix is thus well positioned to participate in the formation of the HR1 coiled coil in the prehairpin intermediate. Env trimer tilting and rearrangements of the α9, MPER and FPPR regions could potentially facilitate the membrane fusion-related functions of the HR2 region, which forms the six-helix bundle with HR1^8,16–18^. Changes in the MPER and FPPR have been shown to destabilize the pretriggered (State-1) Env conformation^11,34^, supporting a role for these elements in maintaining State-1. Thus, rearrangements of the MPER and FPPR could release the restraints that stabilize a State-1 conformation of gp41, allowing transitions to fusion-active conformations.

Only modest differences were detected in the overall antigenicity of the AE2 Env–SMALP complexes, presumably representative of the DIS (State-2), and that of the cleaved, cell-surface AE2 Env, presumably representative of the pretriggered conformation (State-1). Moreover, antibody docking studies suggest that the asymmetric AE2 trimers are nearly comparable to the symmetric SOSIP trimers in discriminating between bNAb and pNAb binding. These results are consistent with previous studies that indicate the limited ability of most bNAbs to distinguish State-1 and State-2 Env conformations^6,32,49^. Nonetheless, interposing a DIS (State-2) between the fully “closed” pretriggered State-1 and CD4-bound “open” conformations (State-3) may confer substantial benefits to HIV-1 with respect to Env immunogenicity. The asymmetry of the DIS (State-2) Env trimer may decrease the elicitation of bNAbs with the correct angles-of-approach needed to bind the pretriggered (State-1) Env trimer; furthermore, bNAbs directed against junctional epitopes (gp120–gp41 hybrid epitopes, V2 quaternary epitopes) may be elicited less efficiently by asymmetric Env trimers. Indeed, some bNAbs (PGT151, PGT145) against these junctional epitopes induce asymmetric Env trimer topologies that differ from the asymmetry observed in the AD8 and AE2 Env trimers; the asymmetric trimers induced by bNAb binding may represent off-pathway, functionally compromised Env conformations.

Even though our efforts did not yield a State-1 Env structure, as plausible models of the DIS, the asymmetric Env trimer structures reported in this study fill gaps in our understanding of HIV-1 entry, Env conformational allostery and antibody evasion. This information can guide the design of therapeutic and prophylactic interventions, as transitions between the pretriggered (State-1) Env and the DIS are modulated positively or negatively by small-molecule inhibitors of HIV-1 entry^5,14^.

## Methods

### Env constructs

The AD8 and AE2 Envs used in this study contain signal peptides and parts of the cytoplasmic tail (residues 751–856) from the HIV-1_HXBc2_ Env^10,34^. Viruses with the AD8 Env exhibit a degree of CD4 dependence and an antibody neutralization profile consistent with those of a primary, Tier-2 HIV-1^10^. The AE2 Env was modified from the AD8 Env to retain greater stability of the functional pretriggered conformation and also contains additional lysine residues to facilitate cross-linking^34^. Compared with the parental HIV-1_AD8_, viruses with the AE2 Env are more resistant to soluble CD4, CD4-mimetic compounds, and cold exposure^34^. The AD8 and AE2 Envs have a Gly–Gly–(His)_6_ tag at the C-terminus of the gp41 cytoplasmic tail.

### Antibodies and CD4-Ig

The antibodies (and their Env epitopes) used in this study include the bNAbs VRC01, VRC03, 3BNC117 and b12 (CD4-binding site, CD4BS); PGT145 and PG9 (V2 quaternary, V2q); PGT151 and 35O22 (gp120–gp41 interface); 2G12 (gp120 outer domain glycans); and 10E8 (gp41 MPER). The pNAbs used in this study include 19b and 39F (gp120 V3); b6 and F105 (gp120 CD4BS); 902090 (gp120 V2 linear); 17b and E51 (gp120 CD4-induced, CD4i); and F240 (gp41 Cluster I). CD4-Ig is a fusion protein consisting of the N-terminal two domains of human CD4 and the Fc portion of an antibody^14^. Antibodies against HIV-1 Env were kindly supplied by Dennis Burton (Scripps), Peter Kwong and John Mascola (Vaccine Research Center, NIH), Barton Haynes (Duke University), Michel Nussenzweig (Rockefeller University), Hermann Katinger (Polymun), James Robinson (Tulane University) and Marshall Posner (Mount Sinai Medical Center). In some cases, anti-Env antibodies were obtained through the NIH AIDS Reagent Program. Antibodies for Western blotting included goat anti-gp120 polyclonal antibody (Thermo Fisher) and the 4E10 anti-gp41 antibody (Polymun). A horseradish peroxidase (HRP)-conjugated rabbit anti-goat antibody (Thermo Fisher) and an HRP-conjugated goat anti-human IgG antibody (Santa Cruz) were used as secondary antibodies for Western blotting.

### BMS-806

BMS-378806 (here called BMS-806) was purchased from Selleckchem.

### Env-expressing cell lines

Human A549 lung epithelial cells (ATCC) inducibly expressing the AD8 and AE2 Envs were established as described^34^. A549-rtTA cells constitutively expressing the reverse Tet transactivator were transduced with an HIV-1-based lentivirus vector expressing Rev and the AD8 or AE2 Envs. The vector transcribes a bicistronic mRNA comprising HIV-1 *rev* and *env* and two selectable marker genes (puromycin and enhanced green fluorescent protein [EGFP]) fused in-frame with a T2A peptide-coding sequence. In the transduced cells, Env expression is controlled by the Tet-responsive element (TRE) promoter and Tet-On transcriptional regulatory elements. Env-expressing cells were enriched by doxycycline induction and fluorescence-activated cell sorting for the coexpressed EGFP marker. Herein, we designate these cells A549-AD8 and A549-AE2, respectively. A549-AD8 and A549-AE2 cultures were adapted for growth in DMEM/F12 medium, supplemented with 10% FBS and penicillin-streptomycin. All cell culture reagents are from Life Technologies.

### Env purification

For the exogenous production of the Env glycoproteins, cells were treated with 2 μg/ml of doxycycline. After 48 hours of culture with doxycycline, the cells were detached with 1x phosphate-buffered saline (PBS) containing 5 mM EDTA and harvested by centrifugation. The remainder of the purification procedure was conducted at 4 °C unless otherwise specified, and 10 μM BMS-806 was added to all buffers. The cell pellets were homogenized in a homogenization buffer (250 mM sucrose, 10 mM Tris-HCl [pH 7.4], 1 mM EDTA, and a cocktail of protease inhibitors [Roche Complete EDTA-free tablets]). Membranes were then extracted from the homogenates by ultracentrifugation. The extracted crude membrane pellet was collected, and resuspended in 1× PBS to a final concentration of 14 mg of wet membrane per ml of 1× PBS. The AD8 Env was purified without cross-linking; membranes with the AE2 Env were cross-linked with 0.35 mM DTSSP for 45 min at 25 °C (Thermo Scientific). Afterward, cell membranes were solubilized with a solubilization buffer containing 100 mM (NH_4_)_2_SO_4_, 20 mM Tris-HCl (pH 8.0), 250 mM NaCl, 20 mM imidazole, 1% (wt/vol) SMA2000 (Gray Valley), and a cocktail of protease inhibitors (Roche Complete EDTA-free tablets). The suspension was ultracentrifuged for 25 min at 100,000 × *g* and 4 °C. The supernatant was collected and mixed with a small volume of preequilibrated Ni-nitrilotriacetic acid (NTA) beads (Qiagen) for 1.5 h on a rocking platform at room temperature. The mixture was then transferred into a small column and washed with a buffer containing 20 mM Tris-HCl (pH 8.0), 100 mM (NH_4_)_2_SO_4_, 1 M NaCl, and 30 mM imidazole. Envs were eluted from the bead-filled column with a buffer containing 20 mM Tris-HCl (pH 8.0), 100 mM (NH4)_2_SO_4_, 250 mM NaCl, and 250 mM imidazole. The eluate containing the AD8 Env was counterselected with the 19b pNAb; the eluate containing the AE2 Env was counterselected with a mixture of the 19b and F240 pNAbs. Counterselection was performed at room temperature by incubation with the antibodies and Protein A-Sepharose beads for 30 min, followed by incubation with Protein A-Sepharose beads alone to remove residual antibodies.

For single-particle cryo-EM analysis, the purified Env–SMALP complexes were dialyzed against a buffer containing 20 mM Tris-HCl (pH 8.0), 100 mM (NH_4_)_2_SO_4_, and 250 mM NaCl. Before cryo-plunging, Cymal-6 (Anatrace) was added to the Env solutions at a final concentration of 0.005%. A 3-µl drop of 0.3 mg/ml Env solution was applied to a glow-discharged UltrAufoil R1.2/1.3 300 mesh Gold grid (Electron Microscopy Sciences), blotted for 2 s, and then plunged into liquid ethane and flash-frozen using an FEI Vitrobot Mark IV.

### Cryo-EM data collection

Cryo-grids were first visually screened on a 200-kV Tecnai Arctica microscope (Thermo Fisher). Qualified grids were then imaged in a 300-kV Titan Krios microscope (Thermo Fisher) equipped with a Gatan BioQuantum energy filter, at a nominal magnification of 105,000 times. Coma-free alignment and parallel illumination were manually optimized prior to data collection. Cryo-EM data for the AD8 Env, including both zero-tilted and 45°-tilted images, were collected on the K2 Summit direct electron detector (Gatan) at a pixel size of 0.685 Å in a super-resolution counting mode, with an accumulated dose of 54 electrons/Å^2^ across 40 frames per movie. A total of 4111 movies were obtained with defocus in the range of –1.0 to –2.7 μm. Cryo-EM data for the AE2 Env, including both zero-tilted and 30°-tilted images, were collected on the K3 Summit direct electron detector (Gatan) at a pixel size of 0.825 Å in a super-resolution counting mode, with an accumulated dose of 51 electrons/Å^2^ across 50 frames per movie. With defocus ranging from –1.0 to –2.7 μm, a total of 11,873 movies were acquired across two sessions.

Both zero-tilted and tilted data were collected through a semi-automatic process set up in SerialEM, as previously described^12^. For zero-tilted movies, the process for movie collection involved four steps: Global focusing and square selection, hole filtration, local focusing for each group of holes, and data acquisition. For tilted movies, each hole’s final xy-coordinates were calibrated according to the stage’s tilting angle, and precise adjustment for defocus was performed for all holes in the first place. Therefore, the stage can be fixed until the end of one round of data acquisition, preventing extra drift due to defocus measurement for new groups.

### Cryo-EM data processing and analysis

Raw movies of each dataset were drift-corrected and dose-weighted at a super-resolution pixel size (0.685 Å for the AE2 dataset, 0.825 Å for the AE2 dataset) in the MotionCor2^50^ program. The first two frames with high drift were discarded before the generation of micrographs. Drift-corrected micrographs were used for the determination of the actual defocus with the Gctf^51^ program. Contaminated or broken micrographs were removed through manual screening.

For the AD8 dataset, particles were automatically recognized and selected from raw micrographs with a modified version of DeepEM^52^, a deep learning-based particle extraction program. For 45°-tilted images, per-particle local CTF estimation was performed using the determined coordinates of particles in Gctf^51^. The resolution used for CTF estimation was confined in Fourier space to a range of 8–20 Å to improve the accuracy. Automatic picking followed by manual examination yielded 365,824 particles for the AD8 Env. 2D classifications were conducted with dose-weighted particles at 2.74 Å/pixel using a box size of 84 × 84 pixels. Two rounds of reference-free 2D classification were performed in ROME^53^, software that combines maximum likelihood-based image alignment and statistical manifold learning-based classification. Bad particles were rejected upon inspection of quality for averaged images of each 2D class, leaving 222,654 particles (119,339 zero-tilted, 103,315 45°-tilted) for 3D analysis.

The initial model for 3D refinement was generated ab initio in RELION 3.0^54^ with particles from diversely oriented 2D classes derived from both the zero-tilted and 45°-tilted datasets and then was low-pass filtered to 80 Å. Unsupervised 3D classification and refinement of the AD8 dataset were performed in RELION 3.0^54^, as summarized in Supplementary Fig. 4a. The first round of 3D classification with global searching was performed in two stages: In the initial 20 iterations, the HealPix order was set at 2 and only signal beyond a resolution of 15 Å was used for the probability calculation; in the subsequent 25 iterations, the HealPix order was enhanced to 3 and the resolution limit was adjusted to 10 Å. Four classes were generated. One class demonstrating a correct trimer shape and abundant secondary structural detail was subjected to a second round of 3D classification with local searching. This class, containing 147,198 particles, was classified into 8 classes under a HealPix of 4 with σ = 4 (meaning that the standard deviation of the Euler angles equals four times the HealPix order). Three favored classes showing high-resolution features were sorted out. The particles contained in these classes were reextracted and binned twofold into 1.37 Å/pixel. Final auto-refinement was conducted in RELION 3.0^54^, applied with a soft-edged global mask when it fell into local searching. One of three reconstructed maps reached the highest resolution of 4.1 Å and is here designated as AD8 Env. According to the in-plane shift and Euler angles of each particle from the final refinement, we reconstructed the two half-maps at a super-resolution counting mode with a pixel size of 0.685 Å. The masked resolutions were measured by gold-standard FSC at a 0.143-cutoff.

For the AE2 dataset, particles were automatically recognized and selected from raw micrographs with a modified version of DeepEM^52^. For 30°-tilted images, per-particle local CTF estimation was performed using the determined coordinates of particles in Gctf^51^. The resolution used for CTF estimation was confined in Fourier space to a range of 8–20 Å to improve the accuracy. Automatic picking followed by manual examination yielded 1,209,493 particles for the AE2 Env, with 876,078 zero-tilted and 333,415 tilted particles. 2D classifications were conducted with dose-weighted particles at 3.3 Å/pixel using a box size of 80 × 80 pixels. Two rounds of reference-free 2D classification were performed in ROME^53^. Bad particles were rejected upon inspection of quality for averaged images of each 2D class, leaving 865,962 (669,049 zero-tilted, 196,913 30°-tilted) particles for 3D analysis.

The initial model for 3D refinement was generated ab initio in RELION 3.0^54^ with particles from diversely oriented 2D classes derived from both the zero-tilted and 30°-tilted datasets, and then was low-pass filtered to 50 Å. Unsupervised 3D classification and refinement of the AE2 dataset were performed in RELION 3.0^51^, as summarized in Supplementary Fig. 4b. The first round of 3D classfication was performed for particles at 3.3 Å/pixel following a two-stage approach similar to that used for the AD8 Env. Two major classes consisting of 52.7% and 46.8% of the input dataset were generated. The 464,217 particles from the larger class with an apparently correct trimer size were reextracted and binned twofold into 1.65 Å/pixel. For this generated dataset, we performed focused 3D classification using subtracted particles, an approach designed to gain more information about the conformations of small subunits with low molecular mass^55^. Specifically, we used Chimera^56^ to segment the map of this generated dataset into a gp120 part and the remaining part, which contains the gp41 ectodomain and the weak density corresponding to the gp41 MPER, TM, and cytoplasmic tail. We subtracted the gp120 part’s projections from the experimental 2D images to get a modified dataset, with CTF corrected and background noise added. The modified particles were then subjected to 3D classification with a soft-edge mask without alignment. 3D classification resulted in 12 classes after 100 iterations, among which four classes demonstrated different extents of density near the MPER region. These four classes were sent for auto-refinement in RELION 3.0^54^ using unmodified particles. Two 3D volumes with higher resolution were generated; these maps, which were obviously distinct, were designated AE2.1 and AE2.2. According to the in-plane shift and Euler angles of each particle from the final refinement, we reconstructed the two half-maps of each state at a super-resolution counting mode with a pixel size of 0.825 Å. The masked resolutions were measured by gold-standard FSC at a 0.143-cutoff.

To explore thoroughly the heterogeneity of AE2 dataset, we retrieved less-favored classes excluded by previous rounds of 3D classification: (1) the second most populated class consisting of 46.8% of the particles from the first round of classification; and (2) the other two major classes from the focused 3D classification that resulted in low-resolution maps after reconstruction. Particles from these classes were combined together and one round of 3D classification was performed with resolution limited beyond 10 Å. One class with 241,694 particles exhibited a map consistent with the structure of the Env trimer and was selected for auto-refinement. However, analysis of its constituent particles indicated a severe anisotropic angular distribution along the central axis that passes through the trimer apex. Most top-view particles were oriented towards one of the protomers. To eliminate such imbalance, we conducted a pseudo-C3 symmetric expansion for this selected dataset. The current 3D volume’s pseudo-C3 symmetric axis was aligned with the main z-axis coordinate. The transformed volume was used as a new initial model to redo auto-refinement for the dataset, in an attempt to align all the particles along the z-axis. After auto-refinement, each particle was rotated 120° around the *z*-axis twice in the same direction. This step enlarged the dataset threefold. We then 3D classified the enlarged dataset into four classes using only local searching, with HealPix order set at 4. As expected, one class with low resolution and three classes in which one conformation faces in three directions were generated. The angular distribution of the particles in the three classes exhibited much-improved isotropy around the symmetric axis. We sorted out one class with the best quality, removed the duplicated particles, and then performed the auto-refinement. The resulting volume turned out to sample an asymmetric conformation identical to that of AE2.1, with a masked resolution of 4.26 Å, measured by gold-standard FSC at a 0.143-cutoff.

### Atomic model building, refinement and visualization

The asymmetric structure of the uncleaved HIV-1_JR-FL_ Env trimer in state U1 with three BMS-806 molecules bound^12^ (PDB: 7N6U) was used as a reference model to build the AD8 and AE2 Env structures. The template structures were docked in Coot^57^, and then main-chain and side-chain fitting was improved manually to generate the starting coordinate files. The fitting of the two models was further improved by real_space_refinement with secondary structure restraints in Phenix^58^. AD8 and AE2 Env glycans were manually refined in Coot with the “Glycan” model, using 7N6U as a reference. Structural comparison was conducted in Pymol and Chimera^56^. All figures of the structures were produced in Pymol or ChimeraX^59^.

### Analysis of AE2 Env glycopeptides

For glycosylation analysis, the AE2 Env was prepared as described above, except that DTSSP cross-linking was omitted. The Env sample was counterselected with the 19b and F240 antibodies followed by incubation with Protein A-Sepharose beads, and directly used (without dialysis) for glycosylation analysis^12,60^. Briefly, the AE2 Env was denatured with urea, reduced with TCEP [Tris (2-carboxyethyl) phosphine hydrochloride] and alkylated with iodoacetamide. The protein was then buffer exchanged and digested with trypsin alone or with a combination of trypsin and chymotrypsin, generating glycopeptides.

The glycopeptides were analyzed by liquid chromatography-mass spectrometry (LC-MS) on an Orbitrap Fusion™ Lumos™ Tribrid (Thermo Scientific) mass spectrometer equipped with ETD (electron transfer dissociation) that was coupled to an Acquity ultra-performance liquid chromatography (UPLC) M-Class system (Waters). About 35 µmol of digest was injected onto a C_18_ PepMap 300 column (300 µm i.d. × 15 cm, 300 Å, Thermo Fisher Scientific) at a flow rate of 3 µL/min. Chromatographic separation was performed using a multi-step gradient with mobile phases consisting of solvent A: 99.9% deionized water + 0.1% formic acid and solvent B: 99.9% acetonitrile + 0.1% formic acid. The gradient started at 3% B for 5 min, followed a linear increase to 40% B in 50 min, then a linear increase to 90% B in 15 min. The column was held at 97% B for 10 min before re-equilibration. The mass spectrometric analysis was performed using data-dependent acquisition with the instrument set to run 3-s cycles for the MS scan at a resolution of 120,000 at *m/z* 400 and two consecutive MS/MS scans with collision-induced dissociation (CID) and ETD. The glycopeptides were identified in the raw data files using a combination of freely available glycopeptide analysis software and expert identification^12,60^.

### Antigenicity of purified AE2 Env–SMALPs

The AE2 Env was purified as described above, except that counterselection with the 19b and F240 pNAbs was omitted. The purified AE2 Env was incubated with antibodies or CD4-Ig together with Protein A-Sepharose beads for one hour at 4 °C. The precipitated Envs were analyzed by Western blotting with a 1:5000 dilution of a goat anti-gp120 polyclonal antibody (Thermo Fisher) and 0.5 μg/ml 4E10 anti-gp41 antibody (Polymun). Secondary antibodies at a 1:5000 dilution were an HRP-conjugated rabbit anti-goat antibody (Thermo Fisher) and an HRP-conjugated goat anti-human IgG antibody (Santa Cruz), respectively.

### Antibody recognition of cell-surface Env

Doxycycline-induced A549-AE2 cells were washed twice with washing buffer (1× PBS plus 5% FBS). Cells were resuspended after treatment with 5 mM EDTA and cross-linked with 0.35 mM DTSSP in the presence of 10 µM BMS-806. BMS-806 was present at 10 µM throughout the rest of the experiment. Aliquots of the cells were incubated with antibodies or CD4-Ig (10 µg/ml) for one hour at 4 °C. After washing in the washing buffer, the cells were lysed in NP-40 lysis buffer (1x PBS, 1% NP-40, 1× protease inhibitor cocktail (Roche)) for 5 min at 4 °C with gentle agitation. The lysates were cleared by centrifugation at 13,200×*g* for 10 min at 4 °C, and the clarified supernatants were incubated with 10 μl Protein A-Sepharose beads (25 mg/ml in PBS) for one hour at room temperature. The beads were pelleted (1000 rpm for 1 min) and washed three times with the final wash buffer (5x PBS, 0.5% NP-40). The beads were suspended in 2x lithium dodecyl sulfate (LDS) sample buffer containing 100 mM DTT, boiled, and analyzed by Western blotting as described above.

For analysis of total Env expression in the cells, samples of the clarified lysates from control cells that had not been incubated with antibodies were saved and analyzed by Western blotting as described above (these are referred to as the “Input” samples).

### Virus neutralization

Single-round recombinant pseudoviruses expressing luciferase were used to measure the inhibition of infection by antibodies and a CD4-mimetic compound, BNM-III-170, as described^34^. Briefly, 293T cells were cotransfected with the Rev/Env-expressing pSVIIIenv plasmid, a Tat-encoding plasmid, the pCMV-Gag-Pol packaging construct, and a plasmid encoding the luciferase-expressing vector. Forty-eight h later, virus-containing supernatants were collected, filtered through a 0.45-μm membrane, and incubated with antibodies or BNM-III-170 for 1 h at 37 °C. The mixture was then added to Cf2Th-CD4/CCR5 cells, which were cultured for 48 h. The cells were lysed, and luciferase activity was measured.

### Statistics and reproducibility

All the measurements were repeated at least three times independently, which showed similar results. The sample size of each measurement was stated in the paper.

### Reporting summary

Further information on research design is available in the Nature Portfolio Reporting Summary linked to this article.

## Supplementary information

Peer Review File

Supplementary Information

Description of Additional Supplementary Files

Supplementary Data

Reporting Summary

## Data Availability

Data supporting the findings of this manuscript are available from the corresponding authors upon reasonable request. The cryo-EM density maps have been deposited in the Electron Microscopy Data Bank (EMDB) under accession codes EMD-28953 (AD8), EMD-28954 (AE2.1), and EMD-28955 (AE2.2). The coordinates have been deposited in the RCSB Protein Data Bank (PDB) under accession codes 8FAD (AD8) and 8FAE (AE2.1). Source data are provided as Supplementary Data.

## References

[1] Wyatt R, Sodroski J (1998). The HIV-1 envelope glycoproteins: fusogens, antigens, and immunogens. Science.

[2] Chen B (2019). Molecular mechanism of HIV-1 entry. Trends Microbiol..

[3] Willey RL, Bonifacino JS, Potts BJ, Martin MA, Klausner RD (1988). Biosynthesis, cleavage, and degradation of the human immunodeficiency virus 1 envelope glycoprotein gp160. Proc. Natl Acad. Sci. USA.

[4] Zhang S (2021). Dual pathways of human immunodeficiency virus type 1 envelope glycoprotein trafficking modulate the selective exclusion of uncleaved oligomers from virions. J. Virol..

[5] Munro JB (2014). Conformational dynamics of single HIV-1 envelope trimers on the surface of native virions. Science.

[6] Herschhorn A (2016). Release of gp120 restraints leads to an entry-competent intermediate state of the HIV-1 envelope glycoproteins. mBio.

[7] Ma X (2018). HIV-1 Env trimer opens through an asymmetric intermediate in which individual protomers adopt distinct conformations. eLife.

[8] Khasnis MD, Halkidis K, Bhardwaj A, Root MJ (2016). Receptor activation of HIV-1 Env leads to asymmetric exposure of the gp41 trimer. PLoS Pathog..

[9] Lu M (2020). Shedding-resistant HIV-1 envelope glycoproteins adopt downstream conformations that remain responsive to conformation-preferring ligands. J. Virol..

[10] Haim H (2011). Contribution of intrinsic reactivity of the HIV-1 envelope glycoproteins to CD4-independent infection and global inhibitor sensitivity. PLoS Pathog..

[11] Wang Q (2022). Global increases in human immunodeficiency virus neutralization sensitivity due to alterations in the membrane-proximal external region of the envelope glycoprotein can be minimized by distant State 1-stabilizing changes. J. Virol..

[12] Zhang S (2021). Asymmetric structures and conformational plasticity of the uncleaved full-length human immunodeficiency virus envelope glycoprotein trimer. J. Virol..

[13] Salimi H (2020). The lipid membrane of HIV-1 stabilizes the viral envelope glycoproteins and modulates their sensitivity to antibody neutralization. J. Biol. Chem..

[14] Si Z (2004). Small-molecule inhibitors of HIV-1 entry block receptor-induced conformational changes in the viral envelope glycoproteins. Proc. Natl Acad. Sci. USA.

[15] Furuta RA, Wild CT, Weng Y, Weiss CD (1998). Capture of an early fusion-active conformation of HIV-1 gp41. Nat. Struct. Biol..

[16] Chan DC, Fass D, Berger JM, Kim PS (1997). Core structure of gp41 from the HIV envelope glycoprotein. Cell.

[17] Weissenhorn W, Dessen A, Harrison SC, Skehel JJ, Wiley DC (1997). Atomic structure of the ectodomain from HIV-1 gp41. Nature.

[18] Melikyan GB (2000). Evidence that the transition of HIV-1 gp41 into a six-helix bundle, not the bundle configuration, induces membrane fusion. J. Cell Biol..

[19] Bonsignori M (2017). Antibody-virus co-evolution in HIV infection: paths for HIV vaccine development. Immunol. Rev..

[20] Ward AB, Wilson IA (2017). The HIV-1 envelope glycoprotein structure: nailing down a moving target. Immunol. Rev..

[21] Kwong PD, Mascola JR (2018). HIV-1 vaccines based on antibody identification, B cell ontogeny, and epitope structure. Immunity.

[22] Sok D, Burton DR (2018). Recent progress in broadly neutralizing antibodies to HIV. Nat. Immunol..

[23] Guttman M (2015). Antibody potency relates to the ability to recognize the closed, pre-fusion form of HIV Env. Nat. Commun..

[24] Sanders RW (2013). A next-generation cleaved, soluble HIV-1 Env trimer, BG505 SOSIP.664 gp140, expresses multiple epitopes for broadly neutralizing but not non-neutralizing antibodies. PLoS Pathog..

[25] Sanders RW (2015). HIV-1 VACCINES. HIV-1 neutralizing antibodies induced by native-like envelope trimers. Science.

[26] Pauthner M (2017). Elicitation of robust Tier 2 neutralizing antibody responses in nonhuman primates by HIV envelope trimer immunization using optimized approaches. Immunity.

[27] Houser KV (2022). Safety and immunogenicity of an HIV-1 prefusion-stabilized envelope trimer (Trimer 4571) vaccine in healthy adults: A first-in-human open-label, randomized, dose-escalation, phase 1 clinical trial. EClinicalMedicine.

[28] Torrents de la Pena A, Sanders RW (2018). Stabilizing HIV-1 envelope glycoprotein trimers to induce neutralizing antibodies. Retrovirology.

[29] Castillo-Menendez LR, Nguyen HT, Sodroski J (2019). Conformational differences between functional human immunodeficiency virus envelope glycoprotein trimers and stabilized soluble trimers. J. Virol..

[30] Cao L (2018). Differential processing of HIV envelope glycans on the virus and soluble recombinant trimer. Nat. Commun..

[31] Torrents de la Pena A (2019). Similarities and differences between native HIV-1 envelope glycoprotein trimers and stabilized soluble trimer mimetics. PLoS Pathog..

[32] Lu M (2019). Associating HIV-1 envelope glycoprotein structures with states on the virus observed by smFRET. Nature.

[33] Lee SC (2016). A method for detergent-free isolation of membrane proteins in their local lipid environment. Nat. Protoc..

[34] Nguyen HT (2022). Functional and highly cross-linkable HIV-1 envelope glycoproteins enriched in a pretriggered conformation. J. Virol..

[35] Julien JP (2013). Crystal structure of a soluble cleaved HIV-1 envelope trimer. Science.

[36] Lyumkis D (2013). Cryo-EM structure of a fully glycosylated soluble cleaved HIV-1 envelope trimer. Science.

[37] Pancera M (2014). Structure and immune recognition of trimeric pre-fusion HIV-1 Env. Nature.

[38] Kwon YD (2015). Crystal structure, conformational fixation and entry-related interactions of mature ligand-free HIV-1 Env. Nat. Struct. Mol. Biol..

[39] Lee JH, Ozorowski G, Ward AB (2016). Cryo-EM structure of a native, fully glycosylated, cleaved HIV-1 envelope trimer. Science.

[40] Pan J, Peng H, Chen B, Harrison SC (2020). Cryo-EM structure of full-length HIV-1 Env bound with the Fab of antibody PG16. J. Mol. Biol..

[41] Rantalainen K (2020). HIV-1 envelope and MPER antibody structures in lipid assemblies. Cell Rep..

[42] Salzwedel K, Berger EA (2000). Cooperative subunit interactions within the oligomeric envelope glycoprotein of HIV-1: functional complementation of specific defects in gp120 and gp41. Proc. Natl Acad. Sci. USA.

[43] Pancera M (2010). Structure of HIV-1 gp120 with gp41-interactive region reveals layered envelope architecture and basis of conformational mobility. Proc. Natl Acad. Sci. USA.

[44] Mangala Prasad V (2022). Cryo-ET of Env on intact HIV virions reveals structural variation and positioning on the Gag lattice. Cell.

[45] Pancera M (2017). Crystal structures of trimeric HIV envelope with entry inhibitors BMS-378806 and BMS-626529. Nat. Chem. Biol..

[46] Chen L (2009). Structural basis of immune evasion at the site of CD4 attachment on HIV-1 gp120. Science.

[47] Pacheco B (2017). Residues in the gp41 ectodomain regulate HIV-1 envelope glycoprotein conformational transitions induced by gp120-directed inhibitors. J. Virol..

[48] Das R, Datta R, Varadarajan R (2020). Probing the structure of the HIV-1 envelope trimer using aspartate scanning mutagenesis. J. Virol..

[49] Cale EM (2022). Antigenic analysis of the HIV-1 envelope trimer implies small differences between structural states 1 and 2. J. Biol. Chem..

[50] Zheng SQ (2017). MotionCor2: anisotropic correction of beam-induced motion for improved cryo-electron microscopy. Nat. Methods.

[51] Zhang K (2016). Gctf: Real-time CTF determination and correction. J. Struct. Biol..

[52] Zhu Y, Ouyang Q, Mao Y (2017). A deep convolutional neural network approach to single-particle recognition in cryo-electron microscopy. BMC Bioinforma..

[53] Wu J (2017). Massively parallel unsupervised single-particle cryo-EM data clustering via statistical manifold learning. PLoS ONE.

[54] Zivanov J (2018). New tools for automated high-resolution cryo-EM structure determination in RELION-3. eLife.

[55] Bai XC, Rajendra E, Yang G, Shi Y, Scheres SH (2015). Sampling the conformational space of the catalytic subunit of human gamma-secretase. eLife.

[56] Pettersen EF (2004). UCSF Chimera–a visualization system for exploratory research and analysis. J. Comput. Chem..

[57] Emsley P, Cowtan K (2004). Coot: model-building tools for molecular graphics. Acta Crystallogr. D. Biol. Crystallogr..

[58] Adams PD (2010). PHENIX: a comprehensive Python-based system for macromolecular structure solution. Acta Crystallogr. D Biol. Crystallogr..

[59] Goddard TD (2018). UCSF ChimeraX: meeting modern challenges in visualization and analysis. Protein Sci..

[60] Go EP (2017). Glycosylation benchmark profile for HIV-1 envelope glycoprotein production based on eleven Env trimers. J. Virol..

[61] Pettersen EF (2021). UCSF ChimeraX: structure visualization for researchers, educators, and developers. Protein Sci..

