# Supplementary material for: Asymmetric conformations of cleaved HIV-1 envelope glycoprotein trimers in styrene-maleic acid lipid nanoparticles
Source: Commun Biol. 2023 May 18;6:535. doi: 10.1038/s42003-023-04916-w (PMC10195785; doi:10.1038/s42003-023-04916-w)
Supplement: Supplementary file 2 — Supplementary Information [file 42003_2023_4916_MOESM2_ESM.pdf]

# Supplementary Information

## Asymmetric conformations of cleaved HIV-1 envelope glycoprotein trimers in styrene-maleic acid lipid nanoparticles

Kunyu Wang, Shijian Zhang, Eden P. Go, Haitao Ding, Wei Li Wang,

Hanh T. Nguyen, John C. Kappes, Heather Desaire, Joseph Sodroski & Youdong Mao

| | |
|---|---|
| Supplementary Table 1 | Pages S2 to S4 |
| Supplementary Table 2 | Page S5 |
| Supplementary Figures 1 through 10 | Pages S6 to S23 |
| Supplementary References 1 through 10 | Page S24 |

**Supplementary Table 1. Antibody docking on an open interface of the asymmetric AD8 Env trimer and on a symmetric sgp140 SOSIP.664 Env trimer (PDB 4ZMJ)[a]**

| bNAb/pNAb | Antibody (PDB ID) | Env Epitope | Env Amino Acid (aa) Residue Clashes[b] | | Env Glycan Clashes[c] | |
|---|---|---|---|---|---|---|
| | | | Asymmetric model (AD8) | Symmetric Model (SOSIP) | Asymmetric model (AD8) | Symmetric Model (SOSIP) |
| bNAb | VRC01 (PDB 5FYK) | CD4BS | No clash with backbone atoms | No clash with backbone atoms | No demonstrated clash | No demonstrated clash |
| bNAb | VRC03 (PDB 3SE8) | CD4BS | No direct clash with backbone atoms; Close proximity to backbone atoms in aa 308, 312-318. | No direct clash with backbone atoms; Close proximity to backbone atoms in aa 204-207, 314-318. | No demonstrated clash | No demonstrated clash |
| bNAb | b12 (PDB 2NY7) | CD4BS | No direct clash with backbone atoms; Close proximity to atoms in aa 206-207, 302-308, 316, 440-441. | Directly clashes with backbone atoms in aa 206-207; Close proximity to atoms in aa 65, 120, 204-205, 208, 303-304, 306-308, 314-318, 437-441. | The tip of glycan on Asn 262; glycan on Asn 301 | The tip of glycan on Asn 262; glycan on Asn 301 |
| pNAb | b13 (PDB 3IDY) | CD4BS | Directly clashes with backbone atoms in aa 300-316; Close proximity to backbone atoms in aa 162-165, 173, 317-324, 437-442. | Directly clashes with backbone atoms in aa 302-308, 437-441; Close proximity to backbone atoms in aa 163-164, 204-207, 298-301, 309-323, 378-379, 436, 442-444. | Glycan on Asn 301 | Glycan on Asn 301 |

| | | | | | | |
|---|---|---|---|---|---|---|
| pNAb | F105 (PDB 3HI1) | CD4BS | Directly clashes with backbone atoms in aa 163-166, 307-317; Close proximity to backbone atoms in aa 120-121, 124, 160-162, 167-171, 206-207, 306, 318. | Directly clashes with backbone atoms in aa 162-166, 306-318; Close proximity to backbone atoms in aa 63-66, 118-123, 159-161 168-173, 202-208, 304-305, 319. | The tip of glycan on Asn 262; glycan on Asn 301 | The tip of glycan on Asn 262; glycan on Asn 301 |
| bNAb | PGT145 (PDB 6NIJ) | Trimer apex | No direct clash with backbone atoms; Close proximity to backbone atoms in aa 162, 166-167. | No direct clash with backbone atoms; Close proximity to backbone atoms in aa 162, 166-169. | No demonstrated clash | No demonstrated clash |
| bNAb | PGT151 (PDB 5FUU) | Gp120-gp41 hybrid | No clash with backbone atoms | No clash with backbone atoms | No demonstrated clash | No demonstrated clash |
| bNAb | PGT121 (PDB 5CEZ) | V3 Glycan | No clash with backbone atoms | No clash with backbone atoms | No demonstrated clash | No demonstrated clash |
| pNAb | 2557 (PDB 3MLR) | V3 crown | Directly clashes with backbone atoms in aa 122-138, 153-176, 186-202, 301-313, 319-324, 423, 435 of the primary docked chain; Directly clashes with backbone atoms in aa 122-130, 160-162, 183-194, 196-198 of the adjacent chain. | Directly clashes with backbone atoms in aa 122-136, 155-176, 187-202, 301-308, 312-313, 319-324 of the primary docked chain; Directly clashes with backbone atoms in aa 122-131, 157-162, 160-162, 182-185, 187-193, 196-198 of the adjacent chain. | No demonstrated clash | No demonstrated clash |

| | | | | | | |
|---|---|---|---|---|---|---|
| pNAb | 3074 (PDB 3MLX) | V3 crown | Multiple direct clashes | Multiple direct clashes | -- | -- |
| pNAb | 17b (PDB 2IFY) | CD4-induced | No direct clash with backbone atoms; Close proximity to backbone atoms in aa 123-127, 160-163, 166-170, 185, 196-200. | Directly clashes with backbone atoms in aa 184-185; Close proximity to backbone atoms in aa 123-127, 160-163, 165-170, 182-183, 187, 192-199. | Glycans on Asn 160, Asn 197 | Glycan on Asn 160 |

[a]Antibodies were docked on an interprotomer interface of the asymmetric AD8 Env trimer with a large opening angle, and on a symmetric sgp140 SOSIP.664 Env trimer (PDB 4ZMJ). The docking of the antibodies was performed as described in the legends to Fig. 7 and Supplementary Figs. 5 and 8.

[b]"Direct clashes" refers to unambiguous overlap between the backbone atoms of the antibody and Env trimer. "Close proximity" indicates that at least one atom of the antibody is within 4 Å of the indicated backbone amino acid (aa) residues on an adjacent protomer of the Env trimer.

[c]Removal of the glycan at Asn 301 has been shown to affect the sensitivity of some HIV-1 strains to neutralization by particular CD4BS antibodies[5-10].

| Env ligand (gp120 epitope) | HIV-1 Env | | | | | |
|---|---|---|---|---|---|---|
| | AD8 | | | AE2 | | |
| | $IC_{50}$ | $IC_{90}$ | $IC_{99}$ | $IC_{50}$ | $IC_{90}$ | $IC_{99}$ |
| VRC03 (CD4BS) | 0.04 | 0.27 | 0.86 | 0.31 | 0.81 | 1.11 |
| VRC01 (CD4BS) | 0.44 | 2.59 | 8.89 | 0.86 | 2.96 | 9.33 |
| b12 (CD4BS) | 1.23 | 11.1 | >20 | 4.89 | >20 | >20 |
| b6 (CD4BS) | >10 | >10 | >10 | >10 | >10 | >10 |
| 19b (V3) | >10 | >10 | >10 | >10 | >10 | >10 |
| 39F (V3) | >10 | >10 | >10 | >10 | >10 | >10 |
| BNM-III-170 (CD4mc-binding site) | 3.01 | 11.1 | 31.1 | 77.8 | >100 | >100 |

**Supplementary Table 2: Inhibition of pseudovirus infection by antibodies and a CD4-mimetic compound.** Recombinant luciferase-expressing HIV-1 pseudotyped by AD8 and AE2 Envs was incubated with a range of concentrations of CD4BS bNAbs (b12, VRC01 and VRC03), a CD4-mimetic compound, BNM-III-170, or the b6, 19b and 39F pNAbs for 1 h at 37 degrees C. The mixtures were incubated with Cf2Th-CD4/CCR5 cells at 37 degrees C and 5% CO2 for 48 h, after which the cells were lysed and luciferase activity was measured. The relative infectivity represents the measured luciferase activity after incubation of the virus with antibodies or BNM-III-170 divided by the activity measured in the absence of antibodies or CD4-mimetic compound. The relative infectivity values as a function of concentration were used to calculate the inhibitory concentrations associated with 50%, 90% and 99% inhibition ($IC_{50}$, $IC_{90}$ and $IC_{99}$, respectively). The values for the antibodies are reported in µg/ml and for BNM-III-170 in µM. The values shown are the means of triplicate samples from a representative experiment. Infection mediated by the AD8 and AE2 pseudotyped viruses was not inhibited by up to 10 µg/ml of the b6 pNAb against the CD4BS or the 19b and 39F pNAbs against the gp120 V3 loop. The experiment was repeated with comparable results.

**a**

| | Env(-) | AD8 | AE2 |
|---|---|---|---|
| **HIV-1 strain of origin** | JR-FL | AD8 | AD8(modified) |
| **Env-producing cells** | CHO | A549 | A549 |
| **Env cleavage** | No | Yes (76%) | Yes (91%) |
| **Ligand** | BMS-806 (added during Env synthesis) | BMS-806 (addded to membrane prep) | BMS-806 (added to membrane prep) |
| **Crosslinker** | BS3 | None | DTSSP |
| **Solubilization** | Cymal-5,A8-35 | SMA | SMA |
| **Counterselection** | None | 19b(V3) | 19b(V3)+F240(gp41) |

**b**

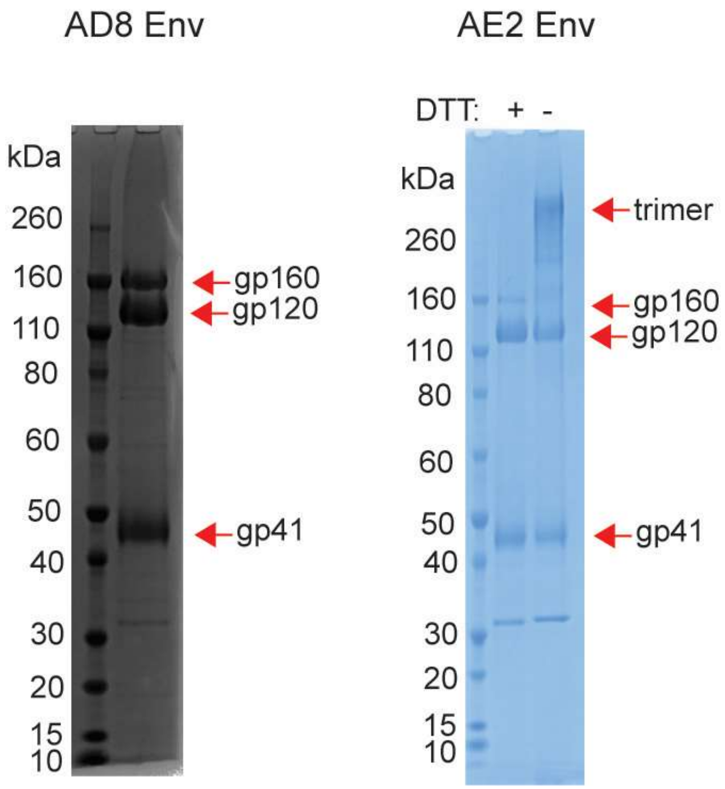


**Supplementary Fig. 1: Preparation of the HIV-1 AD8 and AE2 Env trimers. a** Variables related to the preparation of uncleaved HIV-1 Env(-) trimers[1] and cleaved AD8 and AE2 Env trimers for cryo-EM analysis are listed. **b** The purified AD8 and AE2 Env-SMALPs were analyzed on reducing SDS-polyacrylamide gels (+DTT). The AE2 Env-SMALP complexes were also analyzed under non-reducing conditions (-DTT). The gels were stained with Coomassie Brilliant Blue.

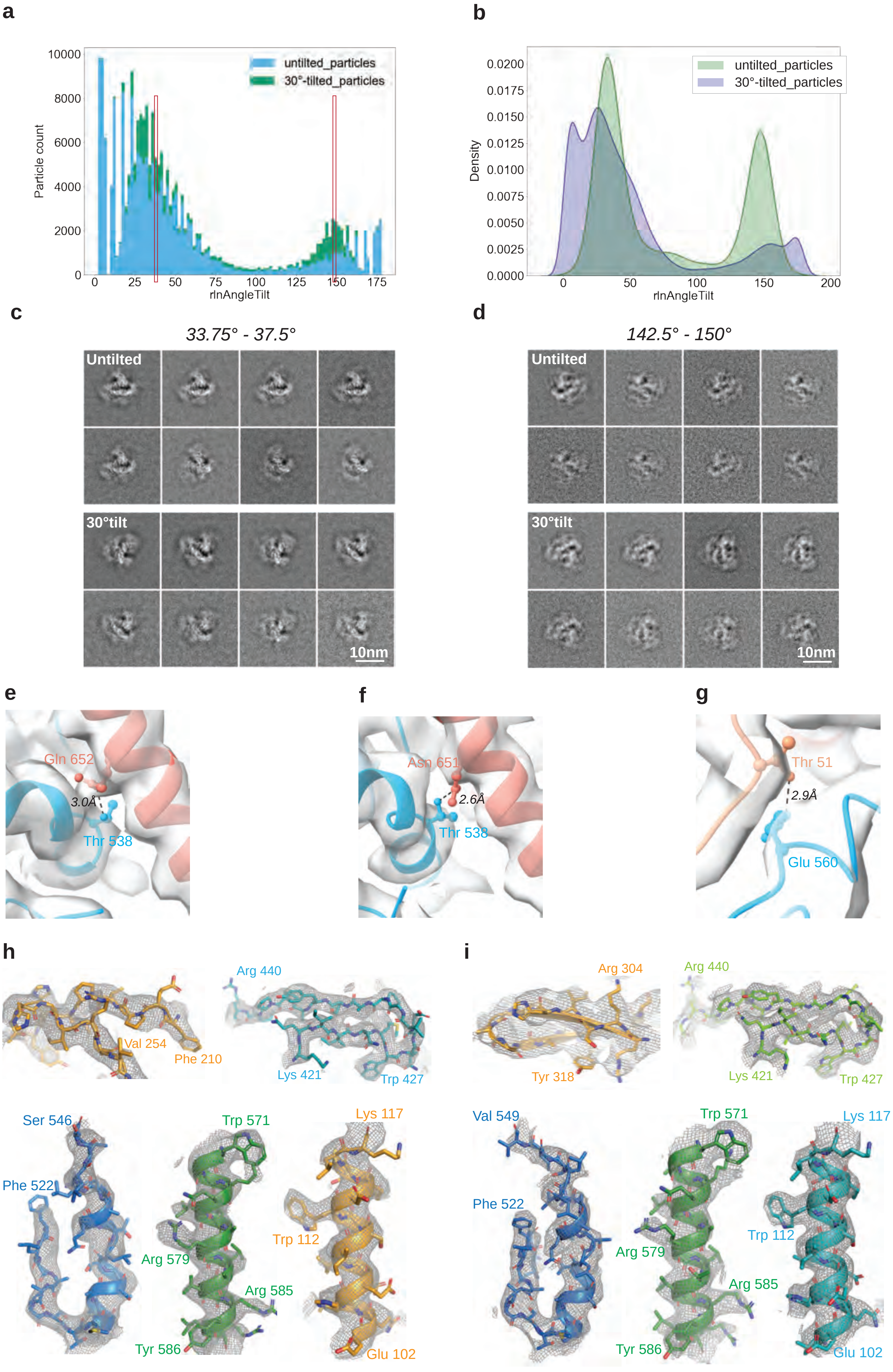
a
b
c
d
e
f
g
h
i
untilted_particles
30°-tilted_particles
Particle count
rlnAngleTilt
Density
33.75° - 37.5°
142.5° - 150°
Untilted
30°tilt
10nm
Gln 652
3.0Å
Thr 538
Asn 651
2.6Å
Thr 51
2.9Å
Glu 560
Arg 440
Val 254
Phe 210
Lys 421
Trp 427
Arg 304
Tyr 318
Ser 546
Phe 522
Trp 571
Arg 579
Arg 585
Tyr 586
Lys 117
Trp 112
Glu 102
Val 549

**Supplementary Fig. 2: Comparison of quality between particles collected at different angles and potential sidechain interactions. a** For an arbitrarily chosen 3D reconstruction of the AE2 Env with resolution exceeding 4.5 Å, particle counts are shown over the whole range (0°-180°) of the second Euler angle (variable name is "rlnAngleTilt" in RELION 3.0). For each bin, particles collected at different tilt angles are colored green (30° tilt) and blue (untilted). **b** The same data as in **(a)**, with the normalized distribution density of the particles collected at different tilt angles plotted across the whole range of the second Euler angle. **c-d** Particles from two angles (indicated by the red boxes in **a**) were extracted for quality examination. The untilted particles and 30° tilt particles were subjected to 2D classification separately. 2D class averages of particles in the range of 33.75° to 37.5° are shown in **c**. 2D class averages of particles in the range of 142.5°to 150° are shown in **d**. **e-g** Some sidechains that may potentially form hydrogen bonds are shown with cryo-EM densities. Distances are measured between the nearest two donor/acceptor atoms**. h** Detailed view of secondary structures in the AD8 Env model and corresponding cryo-EM density. **i** Detailed view of secondary structures in the AE2.1 Env model and corresponding cryo-EM density.

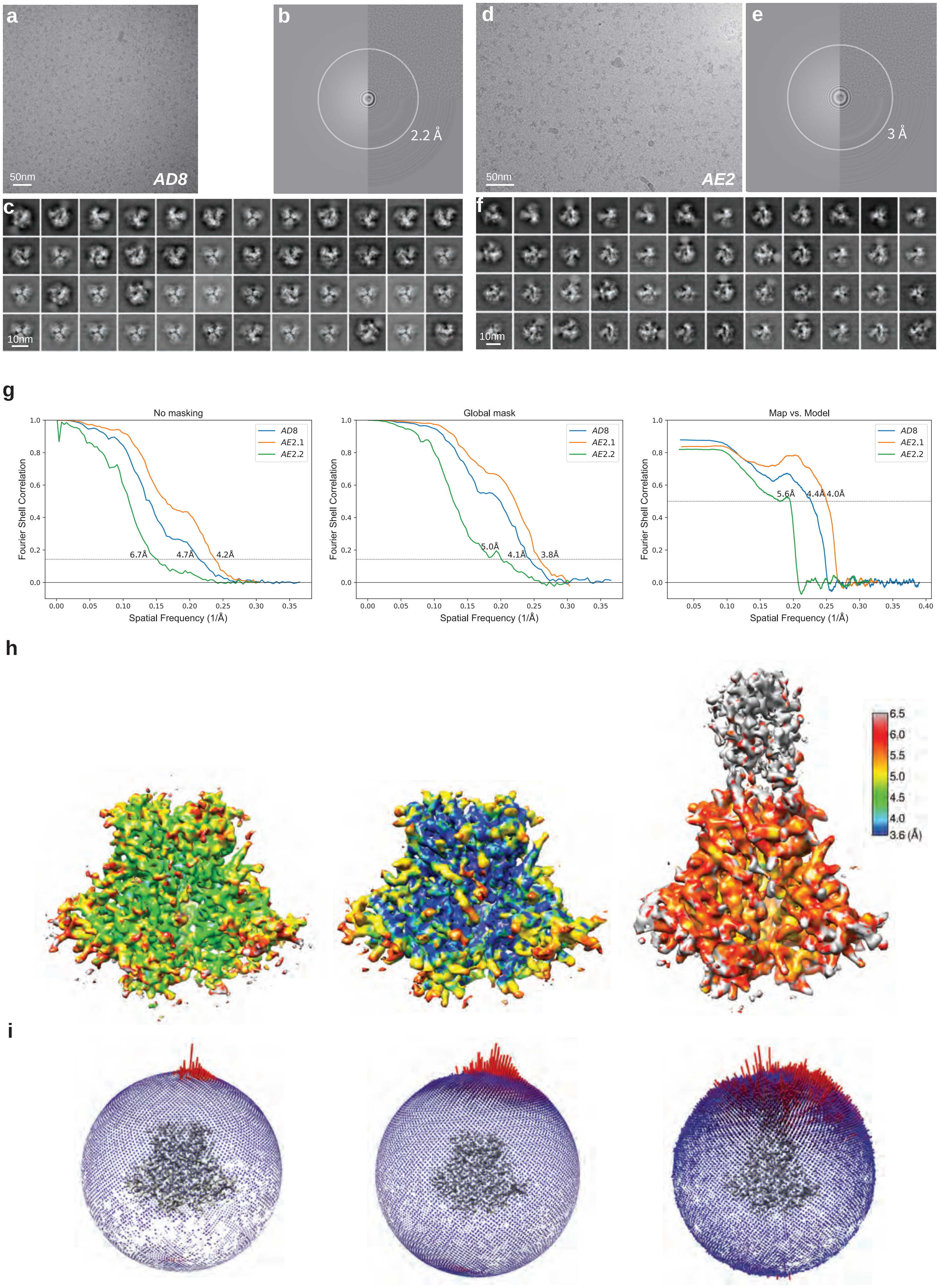
a
50nm
AD8
b
2.2 Å
d
50nm
AE2
e
3 Å
c
10nm
f
10nm
g
No masking
Global mask
Map vs. Model
Fourier Shell Correlation
Spatial Frequency (1/Å)
AD8
AE2.1
AE2.2
6.7Å
4.7Å
4.2Å
5.0Å
4.1Å
3.8Å
5.6Å
4.4Å
4.0Å
h
6.5
6.0
5.5
5.0
4.5
4.0
3.6 (Å)
i

**Supplementary Fig 3: Cryo-EM analysis of the AD8 and AE2 Env trimers. a** A typical cryo-EM micrograph of the AD8 Env trimers. **b** Fourier transform of the micrograph in **a**, generated in Gctf[2]. **c** Selected averaged images of the AD8 Env trimers from unsupervised 2D classification. **d** A typical cryo-EM micrograph of the AE2 Env trimers. **e** Fourier transform of the micrograph in **d**, generated in Gctf[2]. **f** Selected averaged images of the AE2 Env trimers from unsupervised 2D classification. **g** Gold-standard FSC plots of the AD8, AE2.1 and AE2.2 cryo-EM maps. **h** Local resolution measurement of the AD8, AE2.1 and AE2.2 cryo-EM maps, from left to right, by Resmap[3]. Maps are colored according to the local resolution, indicated by the color gradient bar. **i** Orientation distribution of the particles used for reconstruction of, from left to right, the maps of the AD8, AE2.1 and AE2.2 Envs.

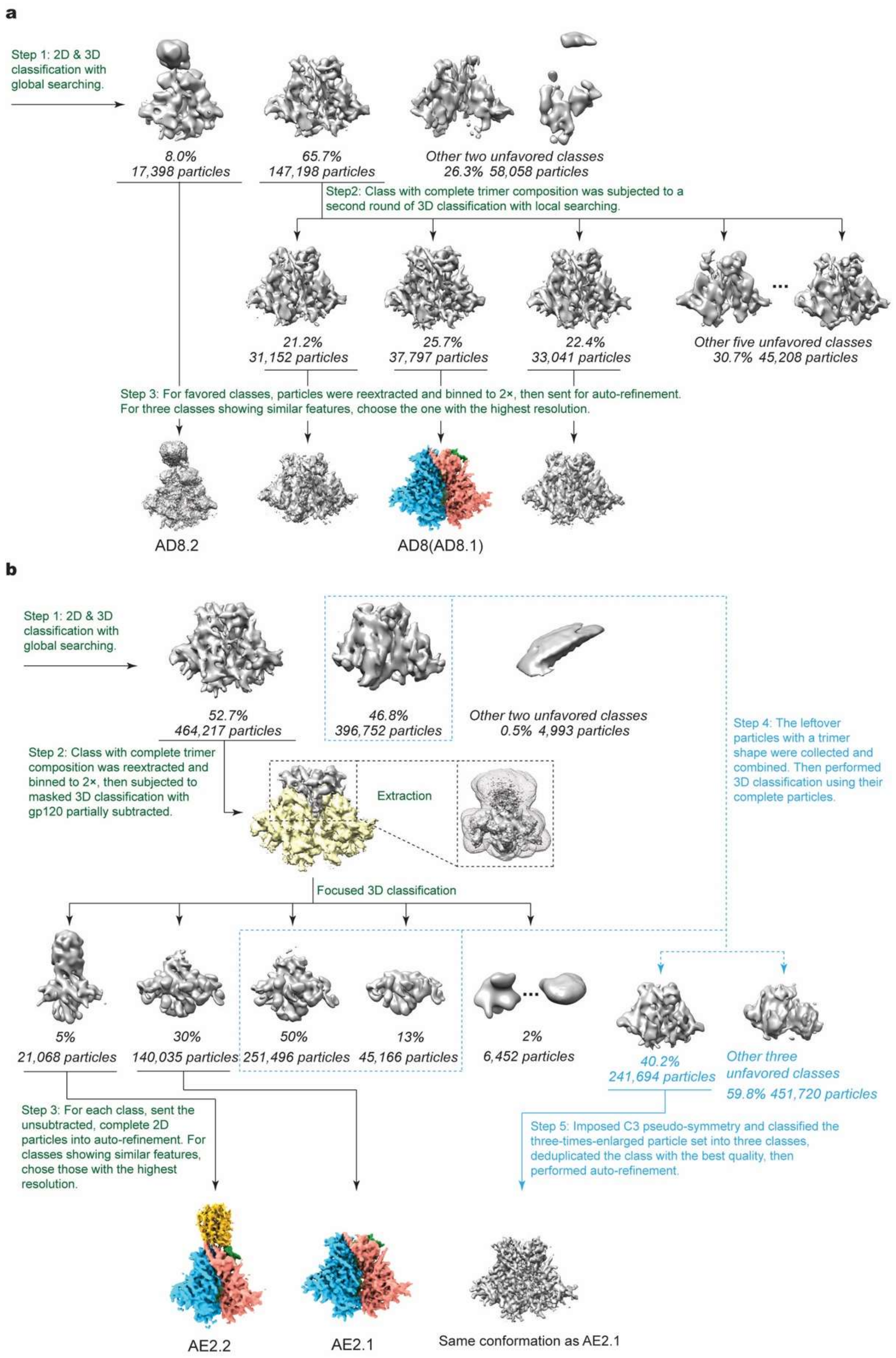
a
Step 1: 2D & 3D classification with global searching.
8.0%
17,398 particles
65.7%
147,198 particles
Other two unfavored classes
26.3% 58,058 particles
Step2: Class with complete trimer composition was subjected to a second round of 3D classification with local searching.
21.2%
31,152 particles
25.7%
37,797 particles
22.4%
33,041 particles
Other five unfavored classes
30.7% 45,208 particles
Step 3: For favored classes, particles were reextracted and binned to 2×, then sent for auto-refinement. For three classes showing similar features, choose the one with the highest resolution.
AD8.2
AD8(AD8.1)
b
Step 1: 2D & 3D classification with global searching.
52.7%
464,217 particles
46.8%
396,752 particles
Other two unfavored classes
0.5% 4,993 particles
Step 2: Class with complete trimer composition was reextracted and binned to 2×, then subjected to masked 3D classification with gp120 partially subtracted.
Extraction
Step 4: The leftover particles with a trimer shape were collected and combined. Then performed 3D classification using their complete particles.
Focused 3D classification
5%
21,068 particles
30%
140,035 particles
50%
251,496 particles
13%
45,166 particles
2%
6,452 particles
40.2%
241,694 particles
Other three unfavored classes
59.8% 451,720 particles
Step 3: For each class, sent the unsubtracted, complete 2D particles into auto-refinement. For classes showing similar features, chose those with the highest resolution.
Step 5: Imposed C3 pseudo-symmetry and classified the three-times-enlarged particle set into three classes, deduplicated the class with the best quality, then performed auto-refinement.
AE2.2
AE2.1
Same conformation as AE2.1

**Supplementary Fig. 4: Cryo-EM data classification workflow. a** The diagram illustrates the major 3D classification steps for the AD8 Env dataset. **b** The diagram illustrates the major 3D classification steps for the AE2 Env dataset.

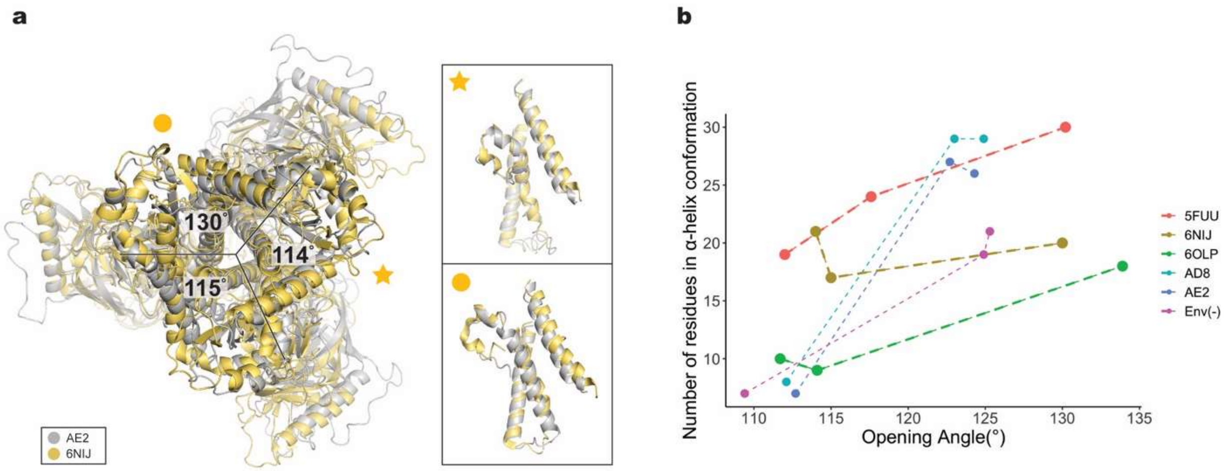


**Supplementary Fig. 5: PGT145-bound Env trimer and relationship between trimer opening angle and FPPR-HR1$_N$ helicity. a** Comparison of the structures of the AE2.1 Env trimer (in grey) and the PGT145-bound full-length HIV-1$_{AMC011}$ Env trimer (PDB 6NIJ) (in yellow). The AE2.1 Env Chain E structure was superposed on Chain E of the PGT145-bound HIV-1$_{AMC011}$ Env structure. The opening angles between the protomers of the PGT145-bound HIV-1$_{AMC011}$ Env trimer are shown. Close-up side views of the interprotomer interfaces with a smaller opening angle (star) and a larger opening angle (circle) are shown in the insets. The close-up views show the gp41 fusion peptide, FPPR, HR1$_N$ and HR1$_C$ regions from the superposed protomers and the α9 helix from the adjacent protomer. **b** The relationship between helicity of the FPPR - HR1$_N$ region and the opening angle of different trimers (AD8, AE2.1, Env(-), PGT145-bound full-length HIV-1$_{AMC011}$ Env trimer (PDB 6NIJ) , PGT151-bound full-length HIV-1$_{AMC011}$ Env trimer (PDB 6OLP) and PGT151-bound HIV-1$_{JR-FL}$ EnvΔCT trimer (PDB 5FUU)) is shown. The x axis represents the opening angle for each of the interprotomer interfaces, measured in PyMOL. The y axis represents the number of residues in an α-helical conformation for the FPPR - HR1$_N$ region (residues Ser 534 – Val 570) associated with an interprotomer interface. The points representing the antibody-bound trimers (5FUU, 6NIJ and 6OLP) are larger to allow them to be distinguished from the Env trimers without protein ligands (AD8, AE2 and Env(-) trimers).

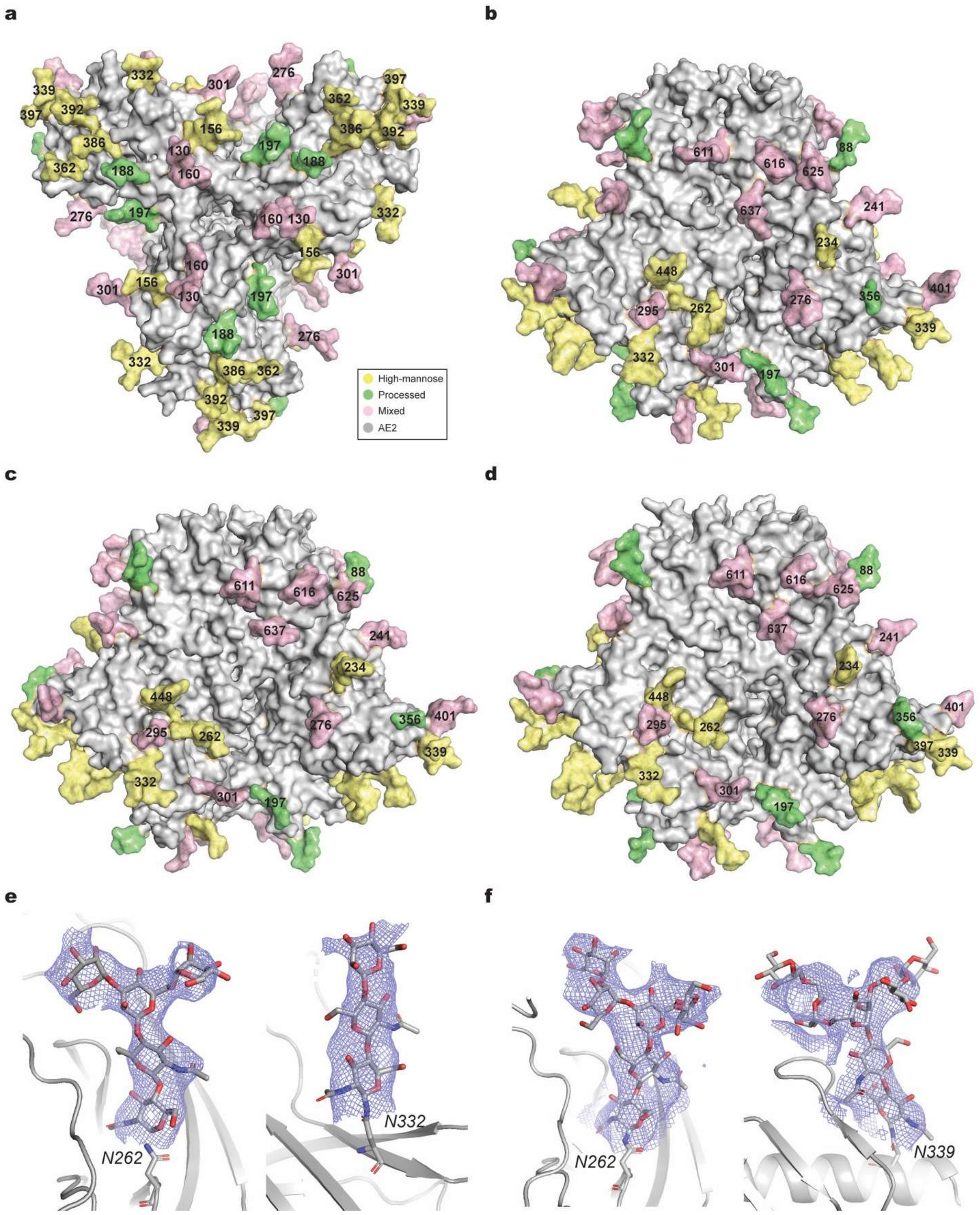


**Supplementary Fig. 6: AE2 Env glycan structure.** Carbohydrates on the map of the AE2.1 Env trimer are colored according to the percentages of glycan types measured by mass spectrometry (Supplementary Fig. 7): high-mannose glycans are colored yellow, processed (complex and hybrid) glycans are colored green, and mixtures of both

types are colored pink. For each glycosylation site, if the probability of either of two glycan types (“High-mannose” or “Processed”) surpasses 80%, then that site’s glycan type is designated as such; otherwise, a “Mixed” glycan type is assigned. **a** View of the AE2.1 trimer from the perspective of the target cell. The interprotomer interface with the smallest opening angle is at the top of the figure. **b** Interface between Chains E, F (left) and Chains A, B (right). This interface has the smallest opening angle. **c** Interface between Chains C, D (left) and Chains E, F (right). **d** Interface between Chains A, B (left) and Chains C, D (right). **e** Detailed view of two “high-mannose” glycans (on Asn 262 and on Asn 332) in the AD8 Env model and their corresponding cryo-EM density. **f** Detailed view of two “high-mannose” glycans (on Asn 262 and on Asn 339) in the AE2.1 Env model and their corresponding cryo-EM density.

**a**

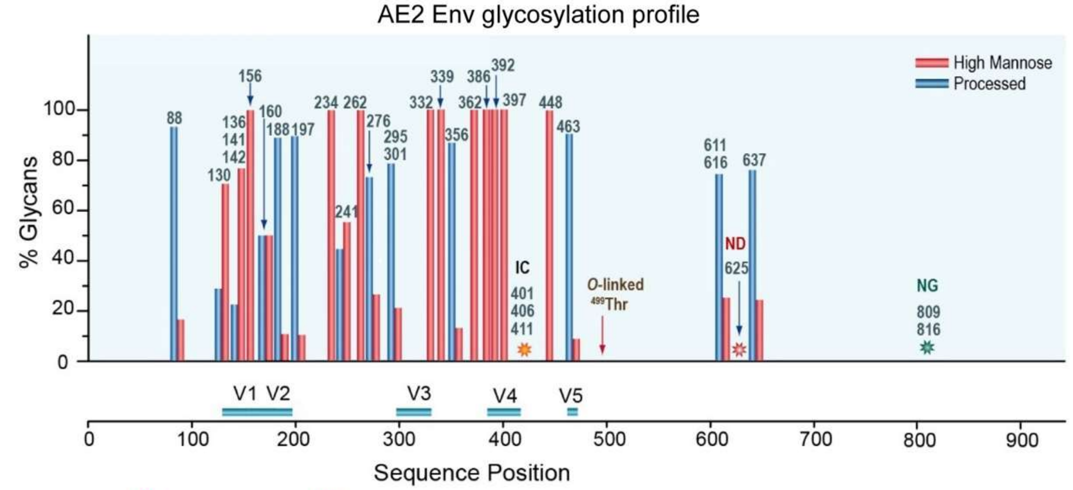


Abbreviations: NG:non-glycosylated; ND: Not Detected in tryptic digests (high-mannose data from Endo H digest); IC: Incomplete

**b**

N-linked glycosylation: 32 sites; O-linked glycosylation: 1* site

| Glycosylated Peptides (*N*-Linked) | Count | %HM | %Proc | %Fuc | %Sia |
|---|---|---|---|---|---|
| ATHACVPTDPNPQEVVLEN$^{88}$VTENF | 31 | 16 | 84 | 58 | 58 |
| LTPLCVTLN$^{130}$CTDLR | 7 | 71 | 29 | 0 | 0 |
| N$^{136}$VTNIN$^{141}$N$^{142}$SSEGMR | 13 | 77 | 23 | 100 | 100 |
| N$^{156}$CSF | 5 | 100 | 0 | 0 | 0 |
| N$^{160}$ITTSIK | 10 | 50 | 50 | 20 | 40 |
| LDVVPIDNDN$^{188}$TSYR | 47 | 11 | 89 | 88 | 86 |
| LINCN$^{197}$TSTITQACPK | 49 | 10 | 90 | 66 | 75 |
| FN$^{234}$GTGPCK | 6 | 100 | 0 | 0 | 0 |
| N$^{241}$VSTVQCTHGIK | 9 | 56 | 44 | 0 | 50 |
| LLN$^{262}$GSLAEEEVVIR | 6 | 100 | 0 | 0 | 0 |
| SSN$^{276}$FTDNAK | 19 | 26 | 74 | 21 | 57 |
| ESVEIN$^{295}$CTRPNN$^{301}$NTR | 33 | 21 | 79 | 65 | 69 |
| QAHCN$^{332}$ISR | 3 | 100 | 0 | 0 | 0 |
| WN$^{339}$NTLNQIATK | 5 | 100 | 0 | 0 | 0 |
| EQFGNN$^{356}$K | 36 | 14 | 86 | 71 | 74 |
| TIVFN$^{362}$QSSGGDPEIVMH | 5 | 100 | 0 | 0 | 0 |
| YCN$^{386}$STQLF | 5 | 100 | 0 | 0 | 0 |
| N$^{392}$STW | 5 | 100 | 0 | 0 | 0 |
| NFN$^{397}$GTW | 5 | 100 | 0 | 0 | 0 |
| N$^{401}$LTQSN$^{406}$GTEGN$^{411}$DTITLPCK* | 22 | Not enough Data | | | |
| CSSN$^{448}$ITGLILTR | 6 | 100 | 0 | 0 | 0 |
| DGGNNHNN$^{463}$DTETFRPGGGDMR | 52 | 10 | 90 | 64 | 92 |
| | | | | | |
| **Transmembrane** | | | | | |
| LICTTAVPWN$^{611}$ASWSN$^{616}$K | 47 | 26 | 74 | 71 | 89 |
| TLDMIWNN$^{625}$MTWMEWEK** | No data from tryptic digest (Endo H data only) | | | | |
| EIDN$^{637}$YTGLIY | 17 | 24 | 76 | 85 | 77 |
| NSAVSLLN$^{816}$ATAIAVAEGTDR | NG | | | | |
| | | | | | |
| Glycosylated Peptides (*O*-Linked) | | | | | |
| IEPLGVAPT$^{499}$K | 2 | | | | |

*Data from Endo H-treated sample and tryptic digest , ** Endo H data only (High-mannose glycans only)

**Supplementary Fig. 7: Glycosylation profile of the purified AE2 Env.** The AE2 Env was prepared in a manner similar to that used to prepare the sample for cryo-EM analysis, except that DTSSP crosslinking was omitted. Glycopeptides were generated and analyzed by mass spectrometry. We identified 32 N-linked glycosylation sites and one O-linked glycosylation site. **a** The glycan composition at each N-linked glycosylation site is shown (high-mannose glycans, red; processed (complex plus hybrid) glycans, blue). **b** The sequences of the glycopeptides are shown, with the modified asparagine residues highlighted in red and the threonine residue modified by O-linked glycans in blue. The percentage of the glycans that are high-mannose (HM), processed (complex and hybrid) (Proc), modified by fucose (Fuc), or sialylated (Sia) are indicated.

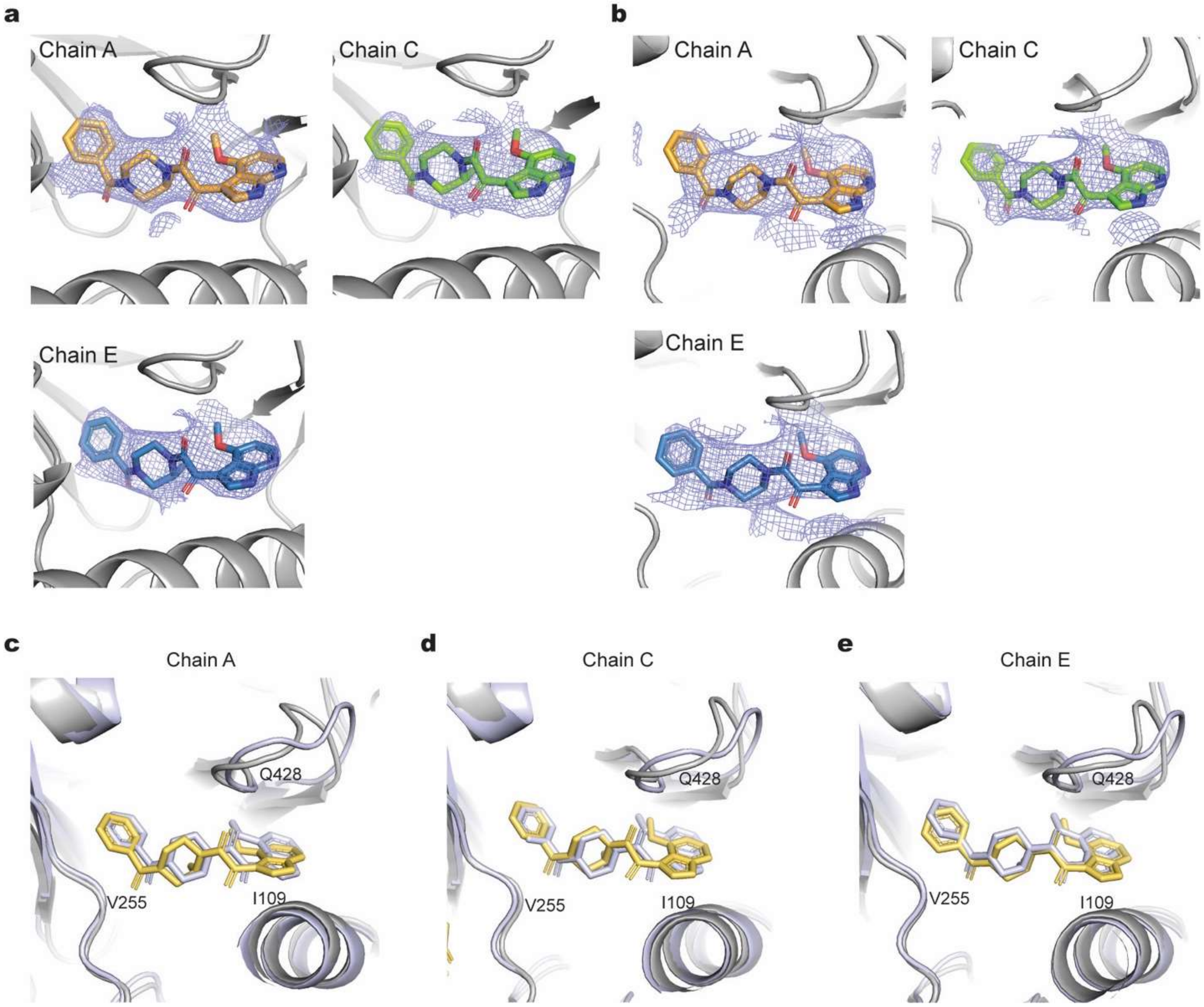


**Supplementary Fig. 8: BMS-806 binding site. a** Detailed views of BMS-806 molecules bound to all three protomers of the AD8 Env model with corresponding cryo-EM density. **b** Detailed views of BMS-806 molecules bound to all three protomers of the AE2.1 Env model with corresponding cryo-EM density. **c-e** The BMS-806 binding sites within the three protomers of the AE2.1 Env trimer (yellow for BMS-806, grey for AE2.1) are compared with those in the BMS-806-bound sgp140 SOSIP.664 trimer (PDB 5U7M) (light purple).[4]

**a**

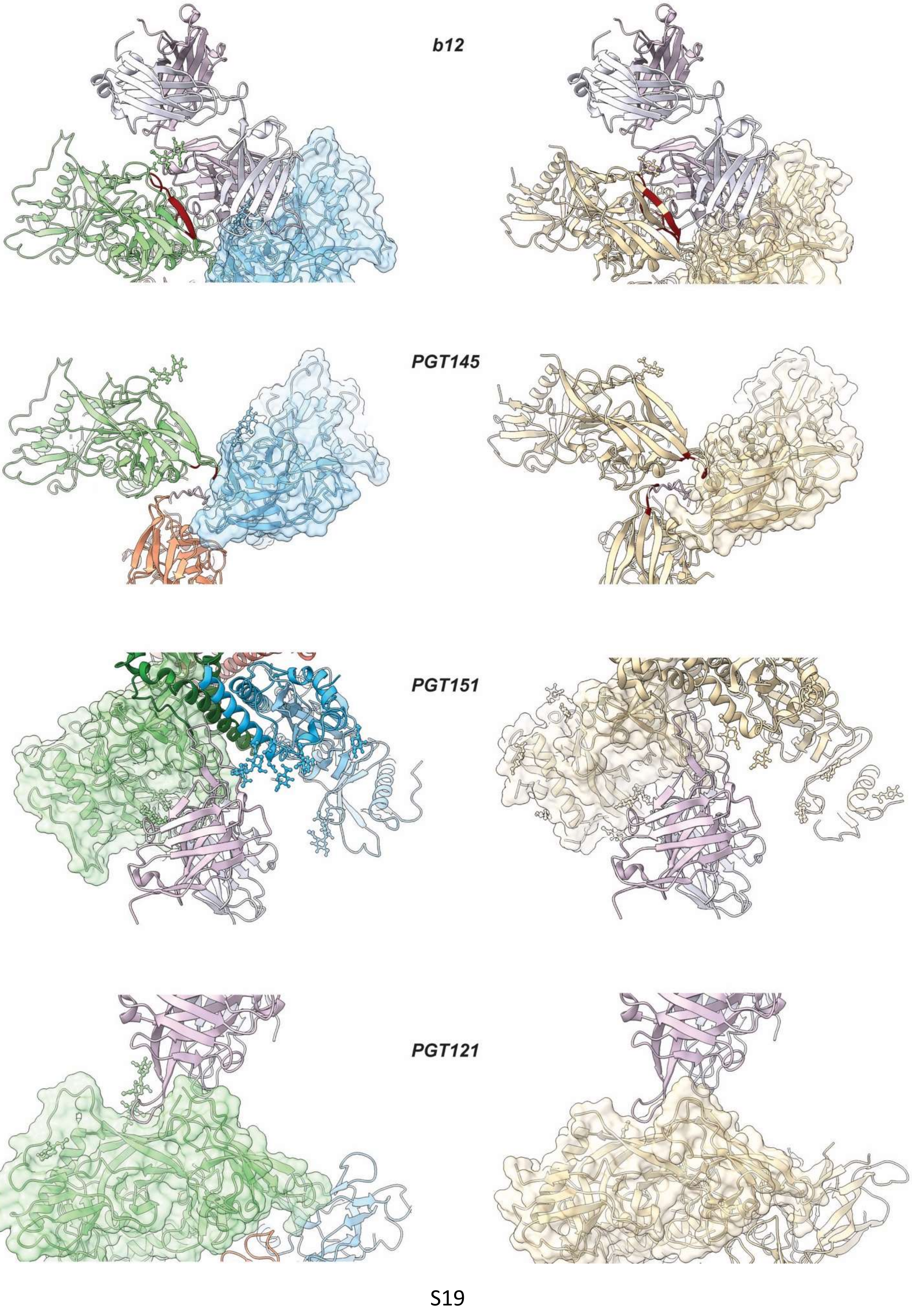

**b**

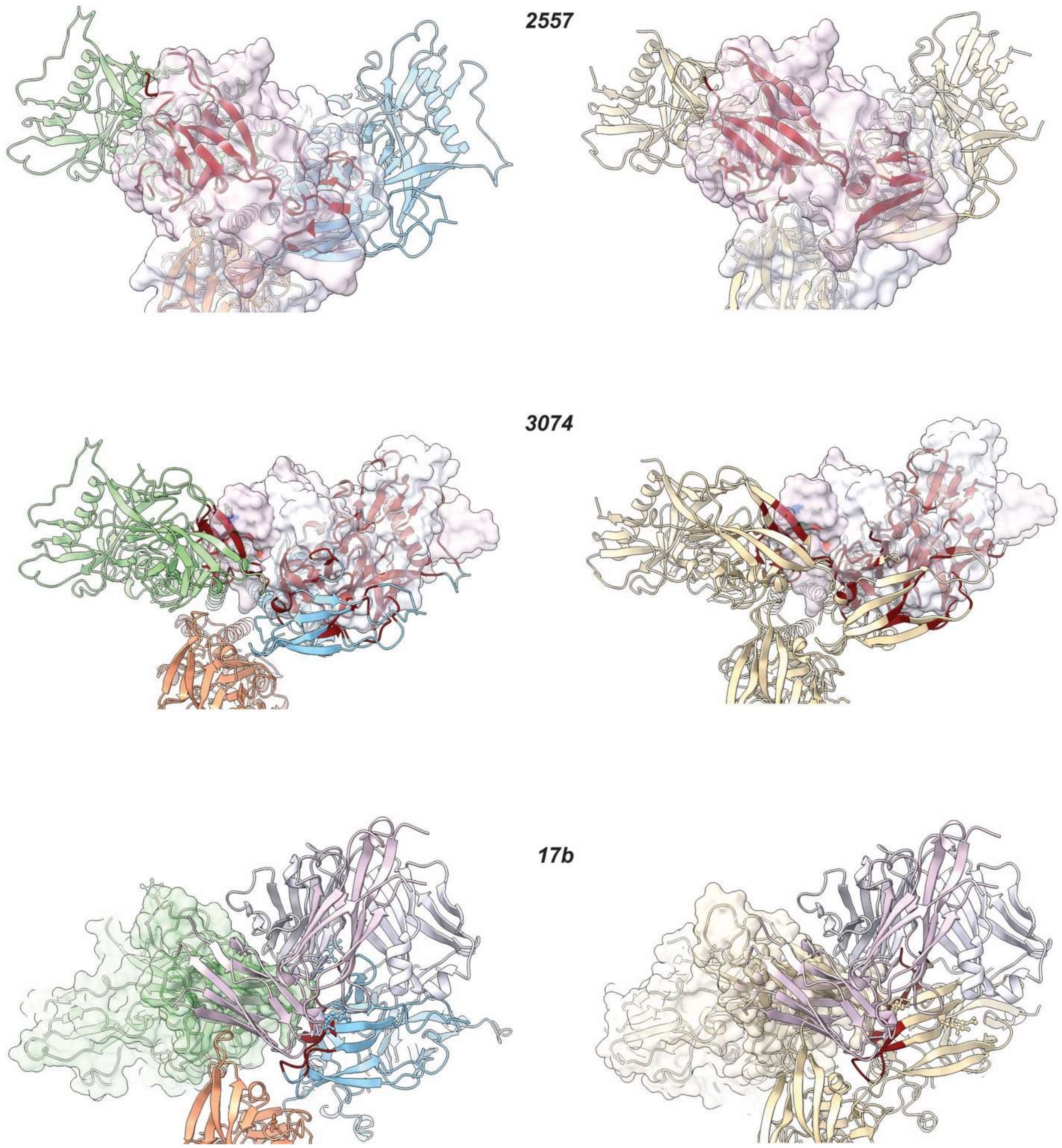


**Supplementary Fig. 9: Antibody docking on a symmetric Env trimer and the more open interface of an asymmetric Env trimer.** Antibody-bound Env trimers or subunits are aligned with the more open interface of the asymmetric AD8 trimer or the symmetric HIV-1$_{BG505}$ SOSIP trimer model (PDB 4ZMJ), as in **Fig. 6**. In **a,** bNAbs are docked; in **b**, pNAbs are docked. For b12 and PGT145, the gp120 of the Env:antibody complex is aligned to Chain E gp120 of the AD8 Env trimer; the rest of the antibody-bound Env trimers are aligned to Chain C gp120 of the AD8 Env trimer that, along with Chain E gp120, flanks an interprotomer interface with a larger opening angle. The molecules are

colored as in **Fig. 6**. The molecular surfaces of the bound 2557 and 3074 anti-V3 antibodies are shown, to help distinguish the antibodies from the Env trimer. The antibodies include: b12 (PDB 2NY7), PGT145 (PDB 6NIJ), PGT151 (PDB 5FUU), PGT121 (PDB 5CEZ), 2557 (PDB 3MLR), 3074 (PDB 3MLX), and 17b (PDB 2I5Y).

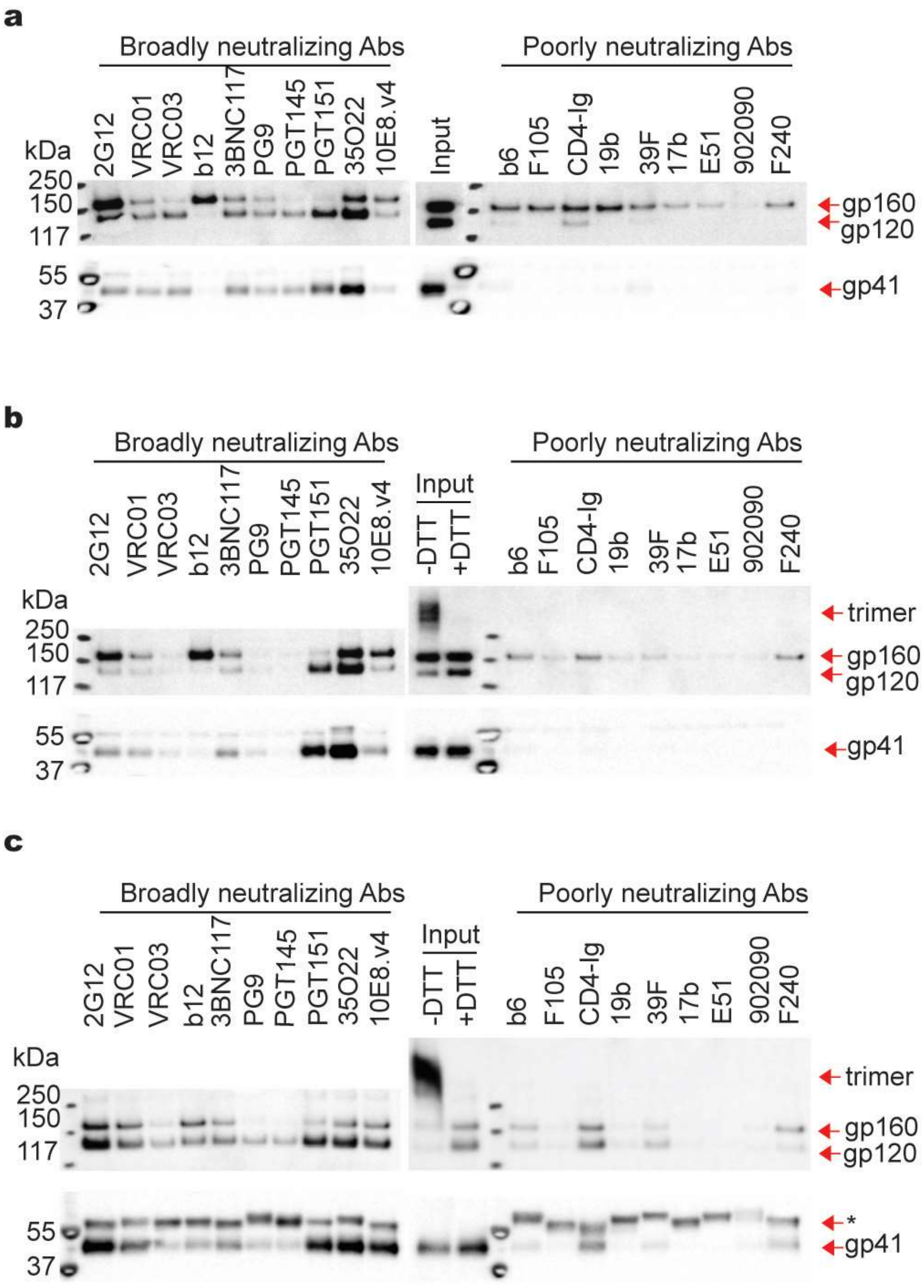
a
Broadly neutralizing Abs
Poorly neutralizing Abs
kDa
250
150
117
55
37
2G12
VRC01
VRC03
b12
3BNC117
PG9
PGT145
PGT151
35O22
10E8.v4
Input
b6
F105
CD4-Ig
19b
39F
17b
E51
902090
F240
gp160
gp120
gp41
b
Broadly neutralizing Abs
Poorly neutralizing Abs
Input
-DTT
+DTT
trimer
gp160
gp120
gp41
c
Broadly neutralizing Abs
Poorly neutralizing Abs
Input
-DTT
+DTT
trimer
gp160
gp120
*
gp41

**Supplementary Fig.10: Antibody recognition of the AE2 Env on the cell surface and in SMALPs. a,b** Recognition of the AE2 Env on the surface of A549 cells by bNAbs, pNAbs and sCD4-Ig. In the experiment shown in **b**, the Env-expressing cells were treated with BMS-806 and crosslinked with DTSSP before incubation with the antibodies or sCD4-Ig; in the experiment shown in **a**, the cells were not treated with BMS-806 or DTSSP. The uncleaved gp160 and cleaved (gp120 and gp41) Envs precipitated by the bound antibodies were detected by Western blotting. **c** Membranes from cells expressing the AE2 Env were treated with BMS-806 and crosslinked with DTSSP. After solubilization with 1% SMA and purification on Ni-NTA beads, the Env-SMALP complexes were incubated with antibodies or sCD4-Ig. The precipitated Envs were analyzed by Western blotting. The gp120/gp160 Western blots are shown in the upper panels. The HRP-conjugated anti-human IgG antibody used as a secondary antibody in the gp41 Western blots (lower panels) detects the heavy chains of the bNAbs, pNAbs and sCD4-Ig (designated with an asterisk).

## Supplementary References


1. Zhang, S. *et al.* Asymmetric structures and conformational plasticity of the uncleaved full-length human immunodeficiency virus envelope glycoprotein trimer. *J Virol* **95**, e0052921 (2021).

2. Zhang, K. Gctf: Real-time CTF determination and correction. *J Struct Biol* **193**, 1-12 (2016).

3. Kucukelbir, A., Sigworth, F. J. & Tagare, H. D. Quantifying the local resolution of cryo-EM density maps. *Nat Methods* **11**, 63-65, doi:10.1038/nmeth.2727 (2014).

4. Pancera, M. *et al.* Crystal structures of trimeric HIV envelope with entry inhibitors BMS-378806 and BMS-626529. *Nat Chem Biol* **13**, 1115-1122 (2017).

5. Xiang, S.-H. *et al*. A V3-dependent gp120 element disrupted by CD4 binding stabilizes the human immunodeficiency virus (HIV-1) envelope glycoprotein trimer. *J Virol* **84**, 3147-3161 (2010).

6. Koch, M. *et al*. Structure-based, targeted deglycosylation of HIV-1 gp120 and effects on neutralization sensitivity and antibody recognition*. Virology* **313**, 387-400 (2003).

7. Malenbaum, S.E. *et al*. The N-terminal V3 loop glycan modulates the interaction of clade A and B human immunodeficiency virus type 1 envelopes with CD4 and chemokine receptors. *J Virol* **74**, 11008-11016 (2000).

8. Gach, JS *et al*. A human antibody to the CD4 binding site of gp120 capable of highly potent but sporadic cross clade neutralization of primary HIV-1*. PLoS One* **8**, e72054 (2013).

9. Wu, X. *et al*. Mechanism of human immunodeficiency virus type 1 resistance to monoclonal antibody b12 that effectively targets the site of CD4 attachment. *J Virol* **83**, 10892-10907 (2009).

10. Powell, R.L.R. *et al*. Plasticity and epitope exposure of the HIV-1 envelope trimer. *J Virol* **91**, e00410-17 (2017).
