# Supplementary material for: Asymmetric conformations of cleaved HIV-1 envelope glycoprotein trimers in styrene-maleic acid lipid nanoparticles
Source: Commun Biol. 2023 May 18;6:535. doi: 10.1038/s42003-023-04916-w (PMC10195785; doi:10.1038/s42003-023-04916-w)
Supplement: Supplementary file 5 — Reporting Summary [file 42003_2023_4916_MOESM5_ESM.pdf]

nature portfolio

Corresponding author(s): Joseph Sodroski, Youdong Mao

Last updated by author(s): May 1, 2023

Reporting Summary

Nature Portfolio wishes to improve the reproducibility of the work that we publish. This form provides structure for consistency and transparency in reporting. For further information on Nature Portfolio policies, see our Editorial Policies and the Editorial Policy Checklist.

Statistics

For all statistical analyses, confirm that the following items are present in the figure legend, table legend, main text, or Methods section.

n/a	Confirmed	
☐	☒	The exact sample size (*n*) for each experimental group/condition, given as a discrete number and unit of measurement
☐	☒	A statement on whether measurements were taken from distinct samples or whether the same sample was measured repeatedly
☒	☐	The statistical test(s) used AND whether they are one- or two-sided<br>*Only common tests should be described solely by name; describe more complex techniques in the Methods section.*
☒	☐	A description of all covariates tested
☐	☒	A description of any assumptions or corrections, such as tests of normality and adjustment for multiple comparisons
☒	☐	A full description of the statistical parameters including central tendency (e.g. means) or other basic estimates (e.g. regression coefficient) AND variation (e.g. standard deviation) or associated estimates of uncertainty (e.g. confidence intervals)
☒	☐	For null hypothesis testing, the test statistic (e.g. *F*, *t*, *r*) with confidence intervals, effect sizes, degrees of freedom and *P* value noted<br>*Give P values as exact values whenever suitable.*
☒	☐	For Bayesian analysis, information on the choice of priors and Markov chain Monte Carlo settings
☒	☐	For hierarchical and complex designs, identification of the appropriate level for tests and full reporting of outcomes
☒	☐	Estimates of effect sizes (e.g. Cohen's *d*, Pearson's *r*), indicating how they were calculated

Our web collection on statistics for biologists contains articles on many of the points above.

Software and code

Policy information about availability of computer code

Data collection	SerialEM v3.6.11
Data analysis	MotionCor2 v1.2.1, GCTF v1.06, DeepEM v1.0, EMAN v2.22, SPIDER v22.10, ROME v1.1.2., RELION v3.1.3., Coot v0.9.5., Phenix v1.19.2, ResMap v1.19.2, Pymal v2.2.3, UCSF Chimera v1.16, ChimeraX v1.2.5

For manuscripts utilizing custom algorithms or software that are central to the research but not yet described in published literature, software must be made available to editors and reviewers. We strongly encourage code deposition in a community repository (e.g. GitHub). See the Nature Portfolio guidelines for submitting code & software for further information.

Data

Policy information about availability of data

All manuscripts must include a data availability statement. This statement should provide the following information, where applicable:

- Accession codes, unique identifiers, or web links for publicly available datasets
- A description of any restrictions on data availability
- For clinical datasets or third party data, please ensure that the statement adheres to our policy

Data supporting the findings of this manuscript are available from the corresponding authors upon reasonable request. The cryo-EM density maps have been deposited in the Electron Microscopy Data Bank (EMDB) under accession codes EMD-28953 (AD8), EMD-28954 (AE2.1), EMD-28955 (AE2.2). The coordinates have been deposited in the RCSB Protein Data Bank (PDB) under accession codes 8FAD (AD8) and 8FAE (AE2.1).

Human research participants

Policy information about studies involving human research participants and Sex and Gender in Research.

Reporting on sex and gender	N/A
Population characteristics	N/A
Recruitment	N/A
Ethics oversight	N/A

Note that full information on the approval of the study protocol must also be provided in the manuscript.

Field-specific reporting

Please select the one below that is the best fit for your research. If you are not sure, read the appropriate sections before making your selection.

☒ Life sciences ☐ Behavioural & social sciences ☐ Ecological, evolutionary & environmental sciences

For a reference copy of the document with all sections, see nature.com/documents/nr-reporting-summary-flat.pdf

Life sciences study design

All studies must disclose on these points even when the disclosure is negative.

Sample size	No statistical methods were used to estimate appropriate sample size.
Data exclusions	No data were excluded from the analysis. During cryo-EM data clustering, good cryo-EM images were chosen for futher 3D analysis based on their achieved resolution and reconstruction quality. Poorer images were excluded in the final reconstructions based on the criteria of maximizing the map resolution and quality.
Replication	All functional experiments were repeated at least three times. All attempts at replication were successful.
Randomization	Single-particle datasets of cryo-EM images were randomly split for the purposes of estimating overall resolution during Fourier shell correlation calculation. Otherwise, randomization was not relevant to these studies.
Blinding	Blinding was not relevant to this study.

Reporting for specific materials, systems and methods

We require information from authors about some types of materials, experimental systems and methods used in many studies. Here, indicate whether each material, system or method listed is relevant to your study. If you are not sure if a list item applies to your research, read the appropriate section before selecting a response.

Materials & experimental systems

n/a	Involved in the study	
☐	☒	Antibodies
☐	☒	Eukaryotic cell lines
☒	☐	Palaeontology and archaeology
☒	☐	Animals and other organisms
☒	☐	Clinical data
☒	☐	Dual use research of concern

Methods

n/a	Involved in the study	
☒	☐	ChIP-seq
☒	☐	Flow cytometry
☒	☐	MRI-based neuroimaging

Antibodies

Antibodies used	The antibodies (and their Env epitopes) used in this study include the bNAbs VRC01, VRC03, 3BNC117 and b12 (CD4-binding site, CD4BS); PGT145 and PG9 (V2 quaternary, V2q); PGT151 and 35O22 (gp120-gp41 interface); 2G12 (gp120 outer domain glycans); and 10E8 (gp41 MPER). The pNAbs used in this study include 19b and 39F (gp120 V3); b6 and F105 (gp120 CD4BS); 902090 (gp120 V2 linear); 17b and E51 (gp120 CD4-induced, CD4i); and F240 (gp41 Cluster I). CD4-Ig is a fusion protein consisting of the N-terminal two domains of human CD4 and the Fc portion of an antibody14. Antibodies against HIV-1 Env were kindly supplied by Dennis Burton

(Scripps), Peter Kwong and John Mascola (Vaccine Research Center, NIH), Barton Haynes (Duke University), Michel Nussenzweig (Rockefeller University), Hermann Katinger (Polymun), James Robinson (Tulane University) and Marshall Posner (Mount Sinai Medical Center). In some cases, anti-Env antibodies were obtained through the NIH ADIS Reagent Program. Antibodies for Western blotting included goat anti-gp120 polyclonal antibody (Thermo Fisher) and the 4E10 anti-gp41 antibody (Polymun). A horseradish peroxidase (HRP)-conjugated rabbit anti-goat antibody (Thermo Fisher) and an HRP-conjugated goat anti-human IgG antibody (Santa Cruz) were used as secondary antibodies for Western blotting.

Validation

Information of the antibody validation is available from manufactuerer's online database or publication. No further validation was done on the antibodies in the reported experiments.

Eukaryotic cell lines

Policy information about cell lines and Sex and Gender in Research

Cell line source(s)	Human A549 lung epithelial cells (ATCC) inducibly expressing the AD8 and AE2 Envs were established as previously described. See Nguyen, H. T. et al. J Virol 96, e0166821 (2022).
Authentication	Further authentication was not performed for this study.
Mycoplasma contamination	Mycoplasma testing was not performed for this study.
Commonly misidentified lines (See ICLAC register)	No commonly misidentified cell lines were used in this study.
