# Supplementary material for: Asymmetric conformations of cleaved HIV-1 envelope glycoprotein trimers in styrene-maleic acid lipid nanoparticles
Source: Commun Biol. 2023 May 18;6:535. doi: 10.1038/s42003-023-04916-w (PMC10195785; doi:10.1038/s42003-023-04916-w)
Supplement: Supplementary file 1 — Peer Review File [file 42003_2023_4916_MOESM1_ESM.pdf]

nature portfolio

Peer Review File

Asymmetric conformations of cleaved HIV-1 envelope glycoprotein trimers in styrene-maleic acid lipid nanoparticles.

Open Access This file is licensed under a Creative Commons Attribution 4.0 International License, which permits use, sharing, adaptation, distribution and reproduction in any medium or format, as long as you give appropriate credit to the original author(s) and the source, provide a link to the Creative Commons license, and indicate if changes were made. In the cases where the authors are anonymous, such as is the case for the reports of anonymous peer reviewers, author attribution should be to 'Anonymous Referee' followed by a clear attribution to the source work. The images or other third party material in this file are included in the article’s Creative Commons license, unless indicated otherwise in a credit line to the material. If material is not included in the article’s Creative Commons license and your intended use is not permitted by statutory regulation or exceeds the permitted use, you will need to obtain permission directly from the copyright holder. To view a copy of this license, visit http://creativecommons.org/licenses/by/4.0/.

This manuscript has been previously reviewed at another Nature Portfolio journal. This document only contains reviewer comments and rebuttal letters for versions considered at Communications Biology.

REVIEWERS' COMMENTS:

Reviewer #2 (Remarks to the Author):

For clarity, we suggest the authors add "Even though our efforts did not yield a State-1 Env structure" in the last paragraph of the discussion.

Point-by-point response to reviewers' comments

We thank all reviewers for taking time to review our manuscript and providing detailed comments.

Reviewer 2

For clarity, we suggest the authors add "Even though our efforts did not yield a State-1 Env structure" in the last paragraph of the discussion.

Response: We have added the suggested statement at the beginning of the last passage of the main text (page 25) accordingly.
